# Naphthalimide-Based Amphiphiles: Synthesis and DFT Studies of the Aggregation and Interaction of a Simplified Model System with Water Molecules

**DOI:** 10.3390/molecules29174204

**Published:** 2024-09-04

**Authors:** Vladislava Petkova, Denitsa Anastasova, Stefan Dobrev, Monika Mutovska, Nikoleta Kircheva, Valya Nikolova, Spas D. Kolev, Stanimir Stoyanov, Yulian Zagranyarski, Todor Dudev, Silvia Angelova

**Affiliations:** 1 Institute of Optical Materials and Technologies “Acad. J. Malinowski”, Bulgarian Academy of Sciences, 1113 Sofia, Bulgaria; vpetkova@iomt.bas.bg (V.P.); sdobrev@iomt.bas.bg (S.D.); nkircheva@iomt.bas.bg (N.K.); sea@iomt.bas.bg (S.A.); 2 Faculty of Chemistry and Pharmacy, Sofia University “St. Kliment Ohridski”, 1164 Sofia, Bulgaria; denii.anastasowaa@gmail.com (D.A.); ohmgm@chem.uni-sofia.bg (M.M.); ohtvd@chem.uni-sofia.bg (V.N.); s.kolev@unimelb.edu.au (S.D.K.); sstoyanov@chem.uni-sofia.bg (S.S.); ohjz@chem.uni-sofia.bg (Y.Z.); 3 Department of Chemical Engineering, School of Chemistry, The University of Melbourne, Melbourne, VIC 3010, Australia; 4 University of Chemical Technology and Metallurgy, 8 St. Kliment Ohridski Blvd, 1756 Sofia, Bulgaria

**Keywords:** naphthalimide, amphiphile, polyethylene glycol, PEG, self-assembly, membrane additive

## Abstract

Systems containing amphiphilic/pathic molecules have the tremendous capacity to self-assemble under appropriate conditions to form morphologies with well-defined structural order (systematic arrangement), nanometer-scale dimensions, and unique properties. In this work, the synthesis of novel naphthalimide-based amphiphilic probes that have 1,8-naphthalimide as the fluorescence signal reporting group, octyl as hydrophobic head, and PEG as hydrophilic tail, is described. These designed molecules represent a new class of self-assembling structures with some promising features. The lack of literature data on the use of 1,8-naphthalimides with cyclic and acyclic hydrophilic PEG fragments as self-assembling structures gives us the opportunity to initiate a new field in materials science. The successful synthesis of such structures is fundamental to synthetic chemistry, and computational studies of the aggregation and binding of water molecules shed light on the ability of these new systems to function as membrane water channels. This study not only expands the list of 1,8-naphthalimide derivatives but may also serve as a new platform for the development of membrane additives based on PEG-functionalized naphthalimides.

## 1. Introduction

Amphiphilic and amphipathic compounds possess both hydrophilic and lipophilic properties. Their molecules contain two distinct covalently bonded components with different solvent affinities—one part has a high affinity for polar solvents (such as water and alcohols) and the other part has a strong affinity for nonpolar solvents, such as hydrocarbons, ethers, and esters, etc. [1]. The tiny difference between amphiphilic and amphipathic molecules is that amphiphilic molecules have less pronounced hydrophilic and hydrophobic regions (portions, sections), whereas amphipathic molecules have a more pronounced separation between hydrophilic and hydrophobic regions (portions, sections). Systems containing amphiphilic/pathic molecules have the tremendous capacity to self-assemble under appropriate conditions to form morphologies with well-defined structural order (systematic arrangement) and nanometer scale dimensions [2]. Self-assembling systems, based on amphiphilic/pathic compounds, find applications in various fundamental and practical fields due to their unique ability to form nano-sized assemblies with gradients of polarity, viscosity, electric charge, and other characteristics [3,4]. The ability to self-assemble into membrane structures even confers a putative role for amphiphilic/pathic compounds in the evolution of membrane structure on early Earth [5,6].

Polyethylene glycols, PEGs (polymers or hydrophilic oligomers generated from ethylene oxide, consisting of -(O-CH_2_-CH_2_)-repeating units), the famous surface and particle modifiers for biological applications, are also known to be amphiphilic. In addition to their hydrophilic property, PEGs also have a hydrophobic character [7]. PEG has important biomedical and biotechnological applications: a chemical modification of bioactive molecules with PEG (or its derivatives) tailors molecular properties to particular applications, eliminating disadvantageous properties or imparting new molecular functions [8]. PEG hydrogels, commonly used in tissue engineering and drug delivery, are water-swollen, three-dimensional, polymer networks that resist protein adhesion and biodegradation [9]. When applied as an additive, PEG acts as a pore-forming agent and increases pore interconnectivity and membrane hydrophilicity [10,11,12,13,14,15]. In turn, the pore structure and hydrophilicity of the membrane play a crucial role in membrane separation processes—a membrane must have good porosity as well as high permeability, hydrophilicity and resistance to impurities (selectivity). The unique properties of PEG provide insight into membrane fusion, a process fundamental to the life of eukaryotic cells [16]. This is due to the ability of PEG to modify cell membranes and mediate cell-to-cell fusion by mediating close contact of the lipid bilayer and inducing evacuation and reorganization of lipid molecules [17]. 

The ability to self-assemble which, as already mentioned, has also been found in amphiphilic compounds, is key to creating ordered supramolecular structures and is used in the development of artificial water channels (AWCs) [18]. One of the main pillars on which the development of the next generation of membranes for water desalination and purification is based is that of AWC [18]. Artificial (or synthetic) channels aim to replicate the exclusive characteristics of transmembrane biological channel proteins, but research in the past has primarily focused on the development of ion channels as a subset of the field of synthetic supramolecular chemistry [19]. Recent findings, after decades of effort, show that it is possible to achieve the performance of benchmark aquaporin (AQP) channels and even to exceed it by new AWC designs using novel features not seen in biology [20].

In the present work, we describe the synthesis of novel amphiphilic naphthalimide-based probes that have 1,8-naphthalimide as the fluorescence signal reporting group, octyl as hydrophobic head, and PEG as the hydrophilic tail. 1,8-Naphthalamides themselves represent an important class of biologically active compounds with favorable photophysical properties, which make them extremely useful bifunctional therapeutic agents and fluorescence imaging agents [21]. Both unsubstituted and substituted naphthalimide units tend to aggregate due to intermolecular π–π interactions among the naphthalimide cores [22,23,24]. In our study, we focus on the core 1,8-naphthalimide fragment and on the PEG hydrophilic tail(s), and substitutions at the nitrogen atom will enable further future functionalization. The octyl chain (hydrophobic head) was reduced to ethyl in order to make theoretical calculations faster. The designed molecules represent a new class of self-assembling structures with some promising characteristics. The lack of literature data on the use of 1,8-naphthalimides with cyclic and acyclic hydrophilic PEG fragments as self-assembling structures gives us the opportunity to pioneer a new field of materials science. The successful synthesis of such structures is fundamental to synthetic chemistry, and the computational study of the aggregation and binding of water molecules are shedding light on the ability of these new systems to function as membrane water channels. This study not only expands the list of 1,8-naphthalamide derivatives but may also serve as a new platform for the development of PEG-functionalized naphthalimide-based membrane additives.

## 2. Results

### 2.1. Synthesis of PEG-Substituted 1,8-Naphthalimides

**Scheme 1:**
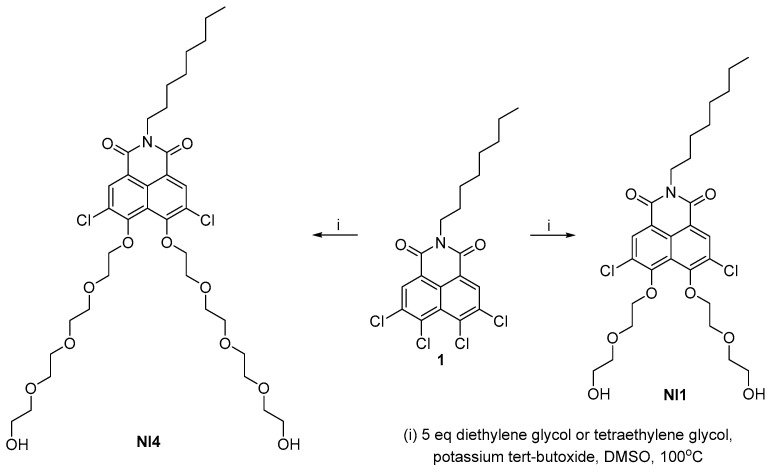
Synthesis of **NI1** and **NI4**.

It is known that alcohols, including polyethylene glycols, are weak nucleophiles and have particular difficultly undergoing nucleophilic aromatic substitution. The deprotonated forms are extremely reactive and although there are few examples in the literature of substitution at the naphthalimide cores, they are suitable nucleophiles [25]. The synthesis of the disubstituted 1,8-naphthalimides with diethylene glycol and tetraethylene glycol is shown in Scheme 1. As a model 1,8-naphthalimide containing two halogen atoms in the *peri*-position, we used *N*-(octyl)-3,4,5,6-tetrachloro-1,8-naphthalimide. Imide **1** was synthesized in two steps in a gram-scale quantity according to our described procedure [26]. The molecule is highly reactive to nucleophilic attack at the *peri*-positions, which is assisted by the imide group as well as the two halogens at positions 3 and 6 [27]. We carried out the nucleophilic aromatic substitution under classical conditions—with DMSO as solvents and potassium *tert*-butoxide as base. Since both reagents are bifunctional, in order to avoid side reactions such as deriving polymer products or products of intramolecular substitution, we used five equivalents of the corresponding glycol. In both cases, the reaction was carried out under moderate heating and a relatively short reaction time. After working up the reaction mixtures, the crude products were purified by column chromatography on silica using dichloromethane/isopropanol mixture as eluent. The naphthalimides **NI1** and **NI4** were isolated in gram scale and with good yields of 53% and 68%, respectively.

**Scheme 2:**
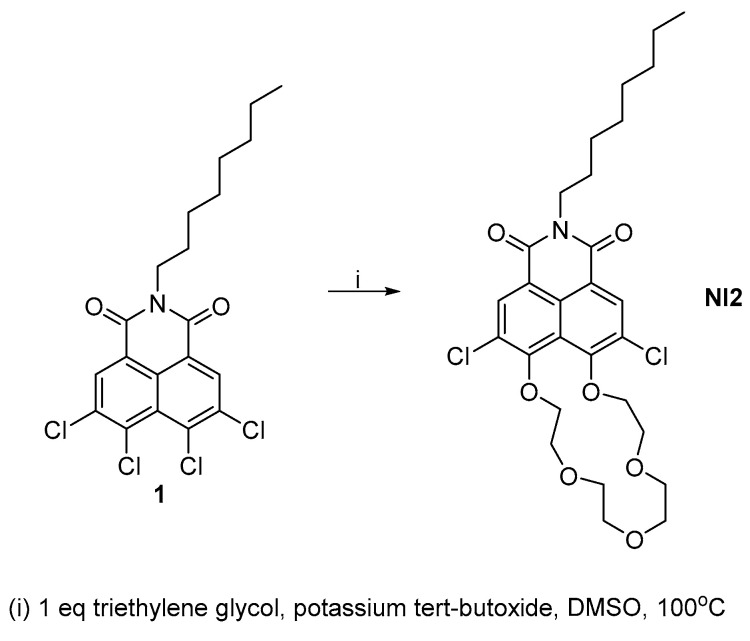
Synthesis of **NI2**.

A similar strategy was used to obtain the crown ether-fused naphthalimide **NI2** (Scheme 2). In order to increase the yield of the intramolecular cyclization product, we used equimolar amounts of the starting imide **1** and triethylene glycol. In addition to this, we conducted the reaction with a significantly lower concentration of reagents compared to the synthesis of **NI1** and **NI4**. The naphthalimides **NI2** was isolated in gram scale and a yield of 25%.

**Scheme 3:**
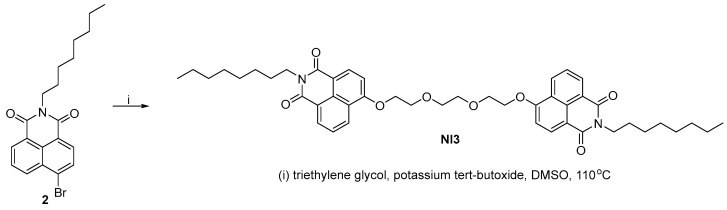
Synthesis of **NI3**.

The synthesis of **NI3** is shown in Scheme 3, using analogous conditions—potassium *tert*-butoxide in DMSO. The reaction was optimized with the highest yield observed at a ratio of starting reagents of 1:2.5 in favor of imide **2** at 110 °C. The crude product was purified by column chromatography on silica and **NI3** was isolated in gram scale with a yield of 71%.

All final products were obtained in good to excellent yields and purity and were characterized by means of ^1^H, ^13^C{1H}-NMR spectroscopic techniques and elemental analysis (see Supplementary Materials).

### 2.2. DFT Studies of the Aggregation and Interaction of PEG-Substituted 1,8-Naphthalimides with Water Molecules

The synthesized PEG-substituted 1,8-naphthalimides represent a new class of supposedly self-assembling structures with intriguing characteristics. Considering the ability of naphthalimides core fragments and (P)EG to aggregate, we decided to computationally model supramolecular structures based on the newly synthesized PEG-substituted 1,8-naphthalimides and investigate their behavior in aqueous solution. 

#### 2.2.1. Water Dimer

Since one of the objects of our study was the interaction with water molecules, a water dimer was chosen for the calibration of the theoretical method. The very first theoretical study of the water dimer was an ab initio calculation by Morokuma and Pedersen published almost six decades ago, in 1968 [28]. Since then, this elementary supramolecular system, which consists of two water molecules loosely bound by a hydrogen bond, has been the focus of unceasing interest of chemists. It is the simplest model system (archetype) for studying hydrogen bonds in water, and like the iconic lab animal the Guinea pig (*Cavia porcellus*), the water dimer has been the subject of so much theoretical (and experimental) research that it has been called “the theoretical Guinea pig” and new ideas and methods are tested on it [29]. 

The focus of this study was not to investigate the different isomers of the water dimer that result from the different possible ways that two water molecules establish a hydrogen bond with each other, but to identify a computational level that reproduces well the energetics of cluster formation. For this reason, the calculations were performed only for the preferred linear non-planar (LNP) isomer of the water dimer [30]. Relevant geometrical (O∙∙∙O distances and O–H∙∙∙O angles) and energetical (ΔE and ΔH) parameters for this isomer, calculated at nine computational levels, are presented in Table 1. The calculations at B3LYP/6-311++G(d,p) level repeat those performed by Ghosh et al. [30] and the obtained value of −5.02 kcal mol^−1^ of interaction energy (basis set superposition error (BSSE)-corrected)) confirms that the optimized geometry of the LNP isomer we obtained is the correct one. A study of the water molecules’ interaction was performed at eight more theoretical levels, and finally, after evaluating the geometrical and energetic parameters and comparing them with those obtained at B3LYP/6-311++G(d,p) level, we judged ωb97xd functional to be the most appropriate. Notably, when the basis set does not include diffuse basis function, the LNP water dimer geometry is not well predicted. The other three basis sets tested gave almost identical results. 

It should be noted that Leforestier et al. also reported a value of 21.0 kJ mol^−1^ (5.02 kcal mol^−1^) for the binding energy, calculated for the water dimer with flexible monomers by applying the ab initio 12-dimensional potential developed by them [31].

The experimentally determined value of the dissociation enthalpy (15.454 ± 0.074 kJ mol^−1^ or 3.694 ± 0.018 kcal mol^−1^) derived by Ruscic [32] was the one that finally guided us to employing the ωb97xd/6-311++G(d,p) combination in subsequent evaluations.

#### 2.2.2. (P)EG/Water

It turns out that PEG hydration and conformation have also been much studied both experimentally and theoretically. PEG repeat units, which we will refer to as EG, are [–CH_2_–CH_2_–O], so (EG)*_n_* is a polymer strand with *n* repeat units. It is known that PEG has almost free rotation around the C–C and C–O single bonds in the gas phase, but this is not the case in the condensed phase when the polymer chain interacts with solvent molecules [33]. Molecular dynamic (MD) simulations of a (EG)_15_ chain reveal a unique pattern for PEG solvated in water: PEG transforms from a collapsed coil to a helical structure; an extended network of PEG–water and water–water hydrogen bonds is found to stabilize the helix backbone [34]. Such a helical structure was also found in the crystalline phase of PEG [35]. The number of water molecules per EG unit ranges from 1 to 6, [34] as the number of water molecules of the first solvation shell per EG unit varies between 0.5 and 1.6 [33].

**Figure 1:**
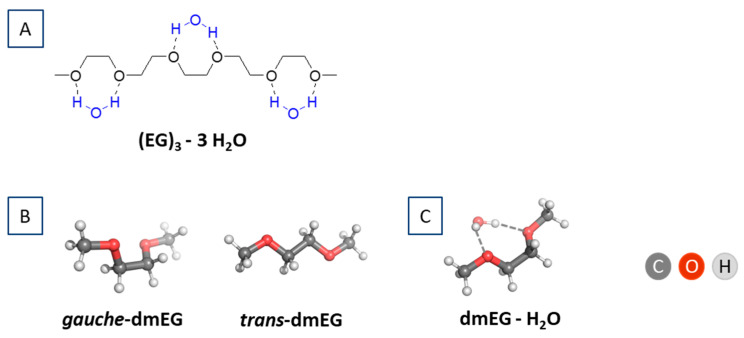
(**A**) (EG)_3_ in gauche conformation with 3 hydrogen-bonded water molecules; (**B**) *Gauche*- and *trans*-dmEG model system; and (**C**) *gauche*-dmEG with one hydrogen-bonded water molecule.

In the next step of our research, to gain insight into the hydration of the PEG, given the complexity of the system and the capabilities of the chosen theoretical method, we used a simple model with one EG unit and methyl terminal groups—dimethylethylene glycol (dimethoxyethane), dmEG (Figure 1). Hydrogen bonds can be formed between the oxygen atoms of EG units and the hydrogen atoms of the water molecule in different positions/geometries. The simplest way is a single hydrogen bond between one of the hydrogen atoms of water with a single oxygen atom of EG. This is the scenario for the *trans* conformers, whereas in the *gauche* conformation, the two oxygen atoms of one EG unit can bond simultaneously to one water molecule (Figure 1). 

Considering the number of water molecules that can be bound by a single EG fragment (between 0.5 and 1.6), and that the energetically favored interaction of water with EG is in a *gauche* conformation, we modeled only a *gauche*-dmEG interaction with a water molecule (Figure 1C).

The associated thermodynamic parameters (BSSE-corrected ΔE and ΔH values) for the hydration reaction of dmEG with a single water molecule in media of different polarity are given in Table 2.

All interactions were favorable and characterized by negative ΔE and ΔH values. With increasing dielectric constants ε from 1 (gas phase) to 78 (water environment) there was a decrease in absolute value of ΔE and ΔH values. The distance between dmEG and the water molecule decreased as the polarity of the medium increased, but this distance remained larger than in a cluster of three water molecules (2.771 Å). ΔE and ΔH values for the gas phase reaction dmEG + H_2_O → [dmEG-H_2_O] were compared with those for the attachment of a water molecule to a water dimer (2H_2_O + H_2_O → [3H_2_O], ΔE = −11.56, ΔH = −9.59 kcal mol^−1^) and the values were found to be smaller (in absolute value).

#### 2.2.3. Naphthalimide-PEG Compounds

##### Aggregation

**Figure 2:**
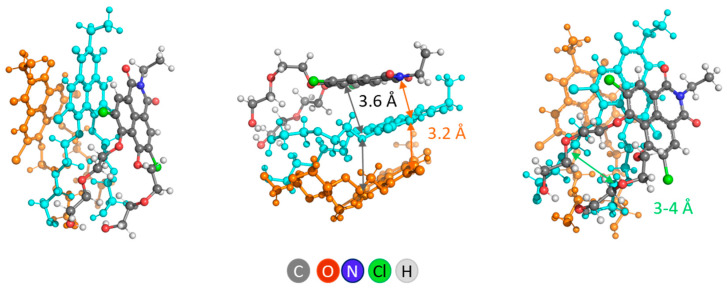
Different views of **3NI1**—the aggregate (cluster) of three **NI1** molecules. For clarity, one of the molecules is orange, another is cyan, and only one is represented with atoms colored by type.

Non-covalent self-association into supramolecular aggregates is the expected behavior of the synthesized PEG-functionalized NIs. The propensity of NI dyes to self-associate was evaluated by modeling aggregates of **NI1**. Based on the theoretical calculations for three **NI1** molecules, we demonstrated that π-stacking is thermodynamically favored, suggesting a preferable structural conformation with parallel displaced **NI1** units (staggered stacking). DFT calculations suggested intermolecular H-bonds between OH groups of PEG tails in a head-to-head disposition of the **NI1** monomers (Figure 2). The distance between layers in this aggregate was about 4 Å, the dihedral angle (angle of divergence) between 2 adjacent molecules was 25°.

The calculations revealed that the formation of such a cluster, composed of 3 **NI1** molecules, is energetically favorable and is characterized by a negative value of ΔH^ε^ (ΔH^1^ = −51.9 kcal mol^−1^ and ΔH^78^ = −40.8 kcal mol^−1^). In this way of arranging the molecules in the cluster (head-to-head staggered stacking mode), there is a channel of small size (3–4 Å) in the fragment, formed by PEG tails.

**Figure 3:**
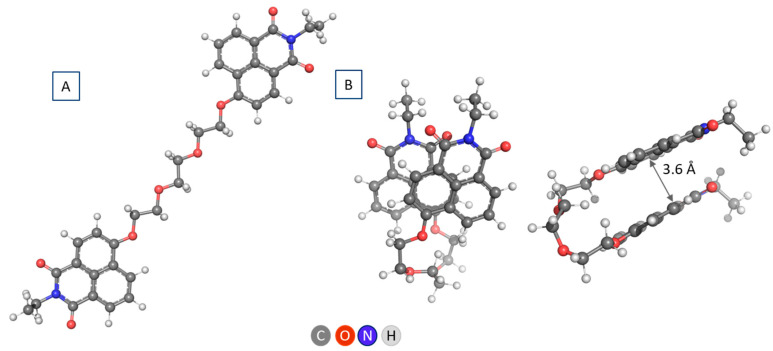
Different views of **NI3** in linear (**A**) and parallel displaced (staggered) conformation (**B**).

In **NI3**, the two naphthalimide fragments of the molecule can be arranged in a similar manner, with the length of the PEG linker sufficient to ensure stacking of the aromatic units (Figure 3). This conformation is preferred over the linear one with 12.9 kcal mol^−1^. The distance between 1,8-naphthalimide layers is of the same size, about 3.6 Å, the dihedral angle (divergence angle) is also smaller, about 20° (Figure 3).

The results obtained for **NI1** and **NI3** are an indicator of the ability of the studied systems to self-associate and/or self-order. The presumed aggregation propensity should also be investigated and confirmed by MD simulations for NIs with PEG tails of different lengths. 

##### Interaction with Water Molecules

**Figure 4:**
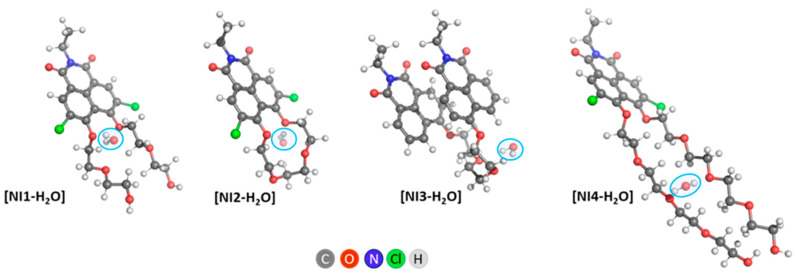
ωb97xd/6-311+G(d,p) optimized [NI-H_2_O] structures. The initial structures subjected to optimization were constructed by placing the explicit water molecule as follows: (1) next to the oxygen atoms closest to the naphthalimide fragment of the EG tail (**NI1** and **NI2**); (2) next to the second and third oxygen atoms of the EG chain (**NI3**); (3) next to the third oxygen atom of the EG chain (**NI4**).

In the present study, our aim was not to systematically investigate the positions at which water molecules can bind, but to establish in principle whether EG tails of PEG-ylated NIs enable such binding. In modeling NI structures with a single water molecule ([NI-H_2_O]), the positions of water relative to NI were sought such that two hydrogen bonds of the water molecule with single oxygen atoms of EG were possible (Figure 4), analogous to the advantageous interaction of the water molecule with *gauche*-dmEG. However, such structures were only obtained for **NI3** and **NI4**.

In **NI1**, the water molecule causes the PEG tails to spread apart and break the hydrogen bond between the hydroxyl groups. Structures are also possible in which the hydrogen bond is retained, and a coordinated water molecule is located in the vicinity of only one EG chain—one such structure is shown in Figure S9. It should be noted that this structure is not as energetically favorable as the one presented in Figure 4. 

**NI2** and **NI3** do not change their conformation when a water molecule is added. In **NI3** we have a *gauche* conformation of an EG fragment, which allows the water molecule to bind to two oxygen atoms from the linker. 

Straight PEG tails of **NI4** are retained upon addition of a water molecule; the water molecule serves as a bridge between the two tails.

The calculated thermodynamic parameters for the water molecule coordination to NIs in solvents with different polarity, characterized by dielectric constants ε from 1 (gas phase) to 78 (water environment) are presented in Table 3. An interesting fact is that EG oligomers and polymers with various numbers of repeat units, which are soluble in water over a wide concentration range, have a strong influence on the dielectric constant of water—it is reduced drastically upon addition of EG/PEG [36]. Some experimental measurements of the static dielectric constant of lower-membered (P)EG have given relatively low values in the order of 10 [37]. For this reason, we considered a medium (implicit solvent) with a dielectric constant of 10, and due to the presence of hydrophobic parts of the NI molecules, we also simulated media with lower ε values of 2 and 5.

All interactions were favorable and characterized by negative ΔE and ΔH values. It should be noted that these results are due to the fact that in modeling the structures of the respective aggregates, positions of the water molecule ensuring its participation in two hydrogen bonds were chosen. 

The energies and enthalpies calculated for the binding process of the water molecule with **NI1** followed the same trend, with ΔH being smaller in absolute values. The values predicted for the reaction **NI1** + H_2_O → [**NI1**-H_2_O] at ε = 2, 5 and 10 are not significantly different. In a more polar medium (water, ε = 78), the absolute values are larger than the values at ε = 2 and ε = 5. The predicted values for the reaction **NI2** + H_2_O → [**NI2**-H_2_O] at ε = 2, 5, and 10 differ, decreasing in absolute value with increasing ε. For the reaction **NI3** + H_2_O → [**NI3**-H_2_O] ΔE and ΔH, values at ε = 1, 2 and 5 are the highest in absolute value; i.e., this reaction proceeds most preferably. The values of ΔE^78^ and ΔH^78^ for this reaction were lower in absolute value than in more nonpolar media (ε = 1, 2 and 5). The elongation of the EG chain was more favorable only in environments with low dielectric constants—this can be seen by comparing the ΔE and ΔH values for **NI1** and **NI4**. 

The O(H_2_O)∙∙∙O(NI) distances remained almost unchanged at different values of ε and were close to the values obtained for dmEG and (H_2_O)_2_.

### 2.3. A Look at Experimental Evidence on Aggregation of NI4 in Water Environment

**Figure 5:**
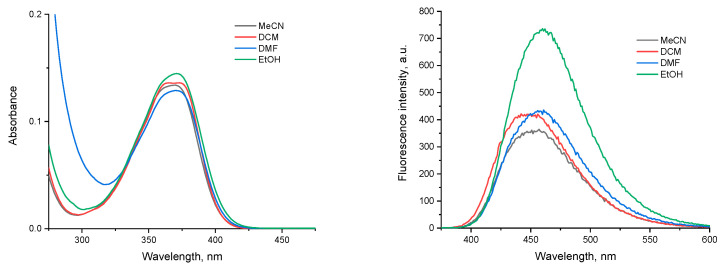
Absorption (**left**) and emission (excitation at 370 nm, **right**) spectra of **NI4** in different solvents (MeCN—acetonitrile, DCM—dichloromethane, DMF—dimethylformamide, EtOH—ethanol), c = 1 × 10^−5^ M.

It was of interest to preliminarily test the spectral properties of the new chromo-phores. As expected, the photophysical characteristics of **NI4**, which was chosen as a representative, were very similar to those of other peri-dialkoxy-substituted naphthalimides, such as Lumogen Violet 570 [38]. The compound showed a broad, structured absorption band in the UV region with a maximum at around 370 nm. The polarity and the proton-donating ability of the solvent does not affect the position and the intensity of the band significantly (Figure 5).

**Figure 6:**
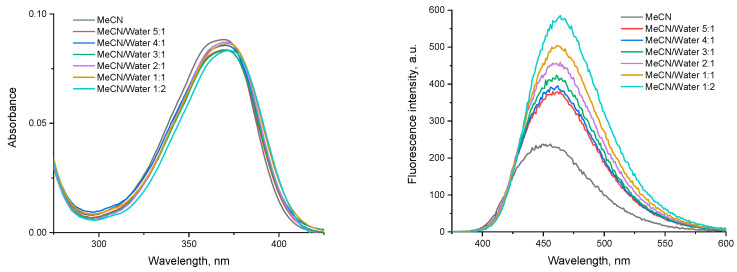
Absorption (**left**) and emission (excitation at 370 nm, **right**) spectra of **NI4** in mixed MeCN/water media.

The fluorescence spectra showed a blue emission with maxima at ca. 450 nm. The bathochromic shift when increasing the solvent polarity clearly indicates a positive solvatochromic effect. The fluorescence intensity is greatly enhanced in protic solvents such as ethanol. To evaluate the spectral properties in water-containing media, a series of samples with the same final concentration of the compound and increasing MeCN to water ratios was prepared and analyzed (Figure 6).

The absorption of **NI4** does not change significantly with the addition of water, but even a small amount of water (MeCN/water 5:1, *v*/*v*) results in bathochromic and hyperchromic shifts of the maximum, maintaining the same trend with increasing water content. Since aggregation-induced emission (AIE) in aqueous media is typical for 1,8-naphthalimide derivatives [23,39,40,41,42,43], it can be assumed that this phenomenon also occurs for **NI4**. 

From the TDDFT calculations performed for **NI4** and [**NI4**-H_2_O] in acetonitrile and water, it turns out that the explicit water molecule plays only a marginal role in the absorption spectra. The maximum absorption wavelengths predicted by TDDFT calculation in acetonitrile (359 nm) and water (365 nm) are in excellent agreement with the experimental measurement (ca. 370 nm). As a result, the adequacy of the chosen computational approach (ωb97xd/6-311+G(d,p) optimized geometry and subsequent TDPBE0/6-311+G(2d,p) single-point calculations, both using implicit solvation in the corresponding solvent) is confirmed. The aggregation processes of the PEG-substituted 1,8-naphthalimides **NI1**–**NI4** will be further investigated in detail by fluorescence spectroscopy and dynamic NMR studies).

## 3. Materials and Methods

All starting materials and solvents were commercially available and used without additional purification after purchase from Fluorochem (Glossop, UK) and Fisher Scientific (Hampton, NH, USA).

NMR spectra were recorded on a Bruker Avance 500 MHz instrument (Bruker, Karlsruhe, Germany) operating at 500 and 126 MHz for ^1^H and ^13^C, respectively. CDCl_3_ was used as solvents. Chemical shifts are reported in δ units (ppm) and referenced to the residual solvent signals (^1^H at 7.26 ppm and ^13^C at 77.160 ppm). Elemental analyses were carried out on a Leco CHNS-932 (Leco Europe, Geleen, The Netherlands). Thin layer chromatographic (TLC) analysis was performed on silica gel plates (Macherey-Nagel F60 254 40 × 80; 0.2 mm, Macherey-Nagel, Duren, Germany) using the solvent system dichloromethane/methanol as an eluent, unless otherwise stated.

### 3.1. Synthesis of **NI1** and **NI4**

General procedure: To a mixture of *N*-(octyl)-3,4,5,6-tetrachloro-1,8-naphthalimide (5.0 mmol, 2.24 g) and corresponding glycol (50 mmol) in 25 mL of DMSO, potassium *tert*-butoxide (11 mmol, 1.23 g) was added. The resulting mixture was stirred for 30 min at 100 °C and then cooled to room temperature. The reaction solution was poured into ice containing 5 mL of concentrated hydrochloric acid. The formed precipitate was filtered, washed thoroughly with water, and dried. The crude product was purified by column chromatography on silica (dichloromethane/isopropanol as eluent) to afford the target compounds as a slightly brownish solid.

**NI1** yield after column chromatography 1.55 g (53%).

^1^H-NMR (δ (ppm), CDCl_3_): 8.60 (s, 2H); 4.35–4.32 (m, 4H); 4.10–4.13 (m, 2H); 3.99–4.01 (m, 4H); 3.77–3.78 (m, 4H); 3.69–3.70 (m, 4H); 2.83 (bs, 2H, OH); 1.64–1.70 (m, 2H); 1.26–1.40 (m, 10H); 0.86 (t, 3H, *J* = 6.9 Hz).

^13^C-NMR (δ (ppm), CDCl_3_): 162.62, 155.65, 133.97, 129.09, 128.40, 123.70, 120.01, 75.47, 73.25, 70.49, 61.95, 40.85, 31.93, 29.42, 29.32, 28.13, 27.18, 22.76, 14.22.

Anal. calcd. C_28_H_37_Cl_2_NO_8_: C, 57.34; H, 6.36; N, 2.39; found: C, 57.11; H, 6.57; N, 2.27.

**NI4** yield after column chromatography 2.59 g (68%).

^1^H-NMR (δ (ppm), CDCl_3_): 8.59 (s, 2H); 4.29–4.32 (m, 4H); 4.09–4.12 (m, 2H); 3.94–3.97 (m, 4H); 3.58–3.71 (m, 24H); 2.42 (bs, 2H, OH); 1.64–1.70 (m, 2H); 1.26–1.40 (m, 10H); 0.86 (t, 3H, *J* = 6.7 Hz).

^13^C-NMR (δ (ppm), CDCl_3_): 162.73, 155.73, 133.89, 128.47, 128.45, 123.62, 119.83, 75.41, 72.71, 70.80, 70.78, 70.73, 70.50, 70.46, 61.86, 40.81, 31.93, 29.42, 29.32, 28.14, 27.19, 22.76, 14.21.

Anal. calcd. C_36_H_53_Cl_2_NO_12_: C, 56.69; H, 7.00; N, 1.84; found: C, 56.43; H, 6.88; N, 2.01.

### 3.2. Synthesis of **NI2**

To a mixture of *N*-(octyl)-3,4,5,6-tetrachloro-1,8-naphthalimide (10.0 mmol, 4.48 g) and triethylene glycol (5 mmol) in 200 mL of DMSO, potassium *tert*-butoxide (22 mmol, 2.46 g) was added. The resulting mixture was stirred for 5 h at 100 °C and then cooled to room temperature. The reaction solution was poured into ice containing 5 mL of concentrated hydrochloric acid. The formed precipitate was filtered, washed thoroughly with water, and dried. The crude product was purified by column chromatography on silica (dichloromethane/isopropanol as eluent) to afford the target compounds as a slightly brownish solid.

**NI2** yield after column chromatography 1.31 g (25%).

^1^H-NMR (δ (ppm), CDCl_3_): 8.60 (s, 2H); 4.35–4.37 (m, 4H); 4.15–4.16 (m, 4H); 4.09–4.13 (m, 2H); 3.87 (s, 4H); 1.64–1.70 (m, 2H); 1.26–1.41 (m, 10H); 0.86 (t, 3H, *J* = 6.9 Hz).

^13^C-NMR (δ (ppm), CDCl_3_): 162.72, 156.49, 134.12, 128.95, 128.37, 124.10, 119.76, 75.10, 71.22, 70.69, 40.81, 31.94, 29.43, 29.32, 28.13, 27.19, 22.76, 14.22.

Anal. calcd. C_28_H_35_Cl_2_NO_7_: C, 59.16; H, 6.21; N, 2.46; found: C, 59.37; H, 6.28; N, 2.31.

### 3.3. Synthesis of **NI3**

To a mixture of *N*-(octyl)-4-bromo-1,8-naphthalimide (7.5 mmol, 2.91 g) and triethylene glycol (3 mmol) in 25 mL of DMSO, potassium *tert*-butoxide (6.6 mmol, 0.74 g) was added. The resulting mixture was stirred for 4 h at 110 °C and then cooled to room temperature. The reaction solution was poured into ice containing 5 mL of concentrated hydrochloric acid. The formed precipitate was filtered, washed thoroughly with water, and dried. The crude product was purified by column chromatography on silica (dichloromethane/isopropanol as eluent) to afford the target compounds as a slightly brownish solid.

**NI3** yield after column chromatography 1.63 g (71%).

^1^H-NMR (δ (ppm), CDCl_3_): 8.51 (dd, 2H, *J*^3^ = 7.3 Hz, *J*^4^ = 1.2 Hz); 8.48 (dd, 2H, *J*^3^ = 8.4 Hz, *J*^4^ = 1.2 Hz); 8.46 (d, 2H, *J*^3^ = 8.3 Hz,); 7.59 (dd, 2H, *J*^3^ = 8.3 Hz, *J*^3^ = 7.3 Hz); 6.97 (d, 2H, *J*^3^ = 8.3 Hz,); 4.39–4.40 (m, 4H); 4.11–4.13 (m, 4H); 4.04–4.06 (m, 4H); 3.85 (s, 4H); 1.67–1.74 (m, 4H); 1.23–1.44 (m, 20H); 0.86 (t, 3H, *J* = 7.0 Hz).

^13^C-NMR (δ (ppm), CDCl_3_): 164.49, 163.93, 159.90, 133.28, 131.55, 129.44, 128.58, 125.93, 123.53, 122.58, 115.47, 106.04, 71.32, 69.68, 68.52, 40.51, 31.97, 29.50, 29.38, 28.31, 27.33, 22.78, 14.23.

Anal. calcd. C_46_H_56_N_2_O_8_: C, 72.23; H, 7.38; N, 3.66; found: C, 72.01; H, 7.29; N, 3.44.

### 3.4. Computational Details

The choice of a reasonable, efficient and yet accurate quantum chemical treatment is a critical step in any computational study [44]. The studied systems (molecules and supramolecular structures) possess single-reference electronic structures and can therefore be described by the common density functional theory (DFT) [45,46] methods. The DFT-based computational protocol includes a thoroughly chosen exchange-correlation energy functional/atomic orbital basis set combination, explicit and implicit solvent treatment and post-optimization CP correction [47] for basis set superposition error (BSSE). 

A consistency check of the selected functional/basis set combination, ωb97XD/6-311+G(d,p) (ωb97XD is a long-range corrected hybrid density functional [48]), was performed by comparing the results of several functionals and basis sets for a representative model system (water dimer, see above). The functionals were selected from the functional class that was conceptually appropriate for the problem under study and the basis sets tested belonged to the Pople family of Gaussian-type contracted basis sets [49]. In fact, the calibration of the theoretical method on the extensively studied water dimer was only part of the selection process. Our choice was guided by the guidelines for proper and effective use of density functionals provided in the works of Mardirossian et al. [50] and Goerigk et al. [51]. The chosen computational method is consistent with the planned calculations of supramolecular structures and with the need to adequately account for non-bonding interactions in the modeled aggregates. ωB97XD, chosen for the geometry optimization, is a long-range corrected hybrid functional that includes empirical dispersion (D) corrections, considered more accurate than commonly used B3LYP for systems where long-range interactions play a significant role, such as non-covalent complexes, organic molecules, and weakly bound systems [48]. ωB97XD belongs to a class of DFT functionals known as range-separated functionals (a subgroup of hybrid functionals), which are capable of capturing both short-range and long-range interactions. 

The same theory level, ωb97XD/6-311+G(d,p), was used for structure optimization and vibrational frequency calculation. A frequency calculation for a fully optimized structure (1) confirms that the obtained structure is a minimum and needs no further refinement, and (2) provides zero-point vibrational energies and thermostatistical corrections to enthalpy.

The solvation model based on the molecular electron density (SMD) [52], a physically complete model, which includes contributions from cavity creation that cost energy in the solvent, and attractive van der Waals interactions with the solvent, was preferred. The energy/enthalpy change (the difference in energies/enthalpies between reactants and products) was used as an indicator to determine whether aggregation or hydration reactions were spontaneous, nonspontaneous, or in equilibrium. 

The enthalpy values for the **NI1** aggregation reaction were also CP-corrected: the CP correction was calculated for **NI1** fragment located in the middle of the cluster, formed by three **NI1** molecules.

The absorption spectra of **NI4** and [**NI4**-H_2_O] in acetonitrile and water were calculated using a time-dependent density-functional theory [53] (TDDFT) method with PBE0 functional (a hybrid functional that contains no parameters that were fitted to experimental data) and 6-311+G(2d,p) basis set. 

All calculations were performed with the Gaussian 09 software package [54]. The PyMOL molecular visualization system Version 2.6 was used for 3D visualization of the optimized molecules and clusters and for preparing high-resolution images [55].

## 4. Conclusions

Synthesis of four novel naphthalimide-based fluorescent amphiphilic probes that have 1,8-naphthalimide as the fluorescence signal reporting group, octyl as hydrophobic head, and PEG as hydrophilic tail, is described in the study herein. A simple method to synthesize these difficult-to-access EG-substituted naphthalimides is proposed. Careful and precise choice of the computational level allowed us to evaluate the ability of EG tails to bind water molecules. The computational results reveal the ability of the studied systems to aggregate into supramolecular structures—this can be inferred from the negative enthalpy values of the reaction to form a supramolecular architecture of three **NI1** molecules and from the energetically preferred parallel displaced (staggered) NI3 conformer. Aggregation of PEG-ylated NI dyes is an important aspect predicted by the performed DFT computations—the propensity to aggregate depends, among other factors, on the molecular structure of the dye: dyes with hydrophilic groups do not aggregate, unlike more hydrophobic dyes. This can be interpreted as a kind of confirmation of the amphiphilic nature of the studied compounds. Regarding the ability of the PEG tails to associate with a water molecule—it was found to be necessary for the EG fragments to be of a particular conformation, so such structural possibilities were sought (and found) for the PEG-substituted NI compounds. The binding of the first water molecule is especially important, as it would serve as a kind of anchor to bind subsequent water molecules. The designed molecules propose a perspective of developing new materials with promising properties. Detailed experimental investigation of the photophysical/chemical properties of the new compounds is envisaged to confirm the theoretically predicted aggregation and interactions with water. The first results on the photophysical/chemical properties of one of the synthesized PEG-substituted NI compounds, **NI4**, are indicative of aggregation-induced emission in aqueous media, which is a typical process for 1,8-naphthalamide derivatives.

## Figures and Tables

**Table 1:** Geometrical and energetic parameters for LNP water dimer; ΔE and ΔH values are in kcal mol^−1^.

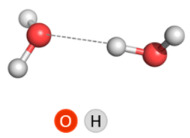	O∙∙∙O Distance, Å	O–H∙∙∙O, °	ΔE	ΔH	ΔE (+BSSE)	ΔH (+BSSE)
B3LYP/6-311++G(d,p) [30]	2.901	175.55	−5.85		−5.05	
B3LYP/6-311++G(d,p)	2.901	174.92	−5.83	−4.08	−5.02	−3.28
B3LYP-D3/6-311++G(d,p)	2.892	174.51	−6.55	−4.78	−5.74	−3.96
B3LYP/6-31G(d,p)	2.878	164.51				
ωb97xd/6-31G(d,p)	2.870	164.17	−7.64	−5.87	−5.65	−3.87
ωb97xd/6-31+G(d,p)	2.871	173.21	−6.45	−4.67	−5.63	−3.85
ωb97xd/6-311+G(d,p)	2.879	174.51	−6.31	−4.54	−5.49	−3.72
ωb97xd/6-311++G(d,p)	2.880	174.76	−6.29	−4.53	−5.45	−3.73
M062X/6-31G(d,p)	2.792	141.54	−7.61	−6.43	−5.20	−4.03
M062X/6-31+G(d,p)	2.880	172.53	−6.64	−4.88	−5.77	−4.01

**Table 2:** ωb97xd/6-311+G(d,p) calculated thermodynamic parameters (in kcal mol^−1^) for the reaction dmEG + H_2_O → [dmEG-H_2_O] in media of different polarity. Mean O(H_2_O)∙∙∙O(dmEG) distances (in Å) are also given.

ε	ΔE (+BSSE)	ΔH (+BSSE)	O(H_2_O)∙∙∙O(dmEG)
1	−8.18	−6.30	2.923
2	−7.86	−5.70	2.898
5	−7.52	−5.20	2.869
10	−6.49	−4.31	2.852
78	−4.25	−2.87	2.824

**Table 3:** ωb97xd/6-311+G(d,p) calculated thermodynamic parameters (in kcal mol^−1^) for the reaction NI + H_2_O → [NI-H_2_O] in media of different polarity. Distances O(H_2_O)∙∙∙O(NI) (in Å) are also given.

ε	ΔE (+BSSE)	ΔH (+BSSE)	O(H2O)∙∙∙O(NI)
**NI1** + H_2_O → [**NI1**-H_2_O]			
1	−4.62	−3.68	2.905
2	−4.30	−3.07	2.891
5	−4.32	−3.25	2.888
10	−4.37	−3.25	2.876
78	−5.67	−4.60	2.871
**NI2** + H_2_O → [**NI2**-H_2_O]			
1	−5.82	−4.22	2.846
2	−4.25	−2.70	2.856
5	−3.45	−1.85	2.869
10	−2.90	−1.41	2.876
78	−3.44	−2.18	2.895
**NI3** + H_2_O → [**NI3**-H_2_O]			
1	−9.93	−8.27	2.875; 2.933
2	−9.00	−6.66	2.867; 2.886
5	−8.25	−6.34	2.854; 2.849
10	−7.38	−5.45	2.841; 2.839
78	−4.96	−3.49	2.828; 2.809
**NI4** + H_2_O → [**NI4**-H_2_O]			
1	−7.19	−5.16	2.892; 2.871
2	−8.00	−4.46	2.887; 2.866
5	−7.55	−3.97	2.882; 2.857
10	−6.58	−2.92	2.875; 2.848
78	−5.01	−2.09	2.828; 2.873

## Data Availability

The data presented in this study are available on request from the corresponding author.

## Supplementary Materials

The following supporting information can be downloaded at: https://www.mdpi.com/article/10.3390/molecules29174204/s1, Figures S1–S8: ^1^H and ^13^C NMR spectra of compounds **NI1**–**NI4**; Figure S9: ωb97xd/6-311+G(d,p) optimized [NI1-H_2_O](a) and [NI1-H_2_O](b) complexes and relative stabilities (expressed as enthalpy difference ΔH); **Table S1**: ωB97XD/6-311+G(d,p) optimized geometries of the studied compounds and/or their aggregates/complexes in the gas phase.
