# Supplementary material for: Naphthalimide-Based Amphiphiles: Synthesis and DFT Studies of the Aggregation and Interaction of a Simplified Model System with Water Molecules
Source: Molecules. 2024 Sep 4;29(17):4204. doi: 10.3390/molecules29174204 (PMC11397715; doi:10.3390/molecules29174204)
Supplement: Supplementary file 1 [file molecules-29-04204-s001.zip › molecules-3107073-supplementary.pdf]

Supplementary Materials

Naphthalimide-Based Amphiphiles: Synthesis and DFT Studies of the Aggregation and Interaction of a Simplified Model System with Water Molecules

Vladislava Petkova [1], Denitsa Anastasova [2], Stefan Dobrev [1], Monika Mutovska [2], Nikoleta Kircheva [1], Valya Nikolova [2], Spas D. Kolev [2,3], Stanimir Stoyanov [2], Yulian Zagranyarski [2], Todor Dudev [2,*], Silvia Angelova [1,4]

[1] *Institute of Optical Materials and Technologies "Acad. J. Malinowski", Bulgarian Academy of Sciences, 1113 Sofia, Bulgaria*

[2] *Faculty of Chemistry and Pharmacy, Sofia University "St. Kliment Ohridski", 1164 Sofia, Bulgaria*

[3] *Department of Chemical Engineering, School of Chemistry, The University of Melbourne, Victoria 3010, Australia*

[4] *University of Chemical Technology and Metallurgy, 8 St. Kliment Ohridski Blvd, 1756 Sofia, Bulgaria*

Contents

Figure S1: ^{1}H NMR spectrum of the compound **NI1.** 2

Figure S2: ^{13}C NMR spectrum of the compound **NI1**. 3

Figure S3: ^{1}H NMR spectrum of the compound **NI2.** 4

Figure S7: ^{1}H NMR spectrum of the compound **NI4.** 8

Figure S8: ^{13}C NMR spectrum of the compound **NI4** 9

Figure S9: ωb97xd/6-311+G(d,p) optimized [NI1-H_2O](a) and [NI1-H_2O](b) complexes and relative stabilities (expressed as enthalpy difference ΔH). 10

Table S1: ωB97XD/6-311+G(d,p) optimized geometries of the studied compounds and/or their aggregates/complexes in the gas phase. 11

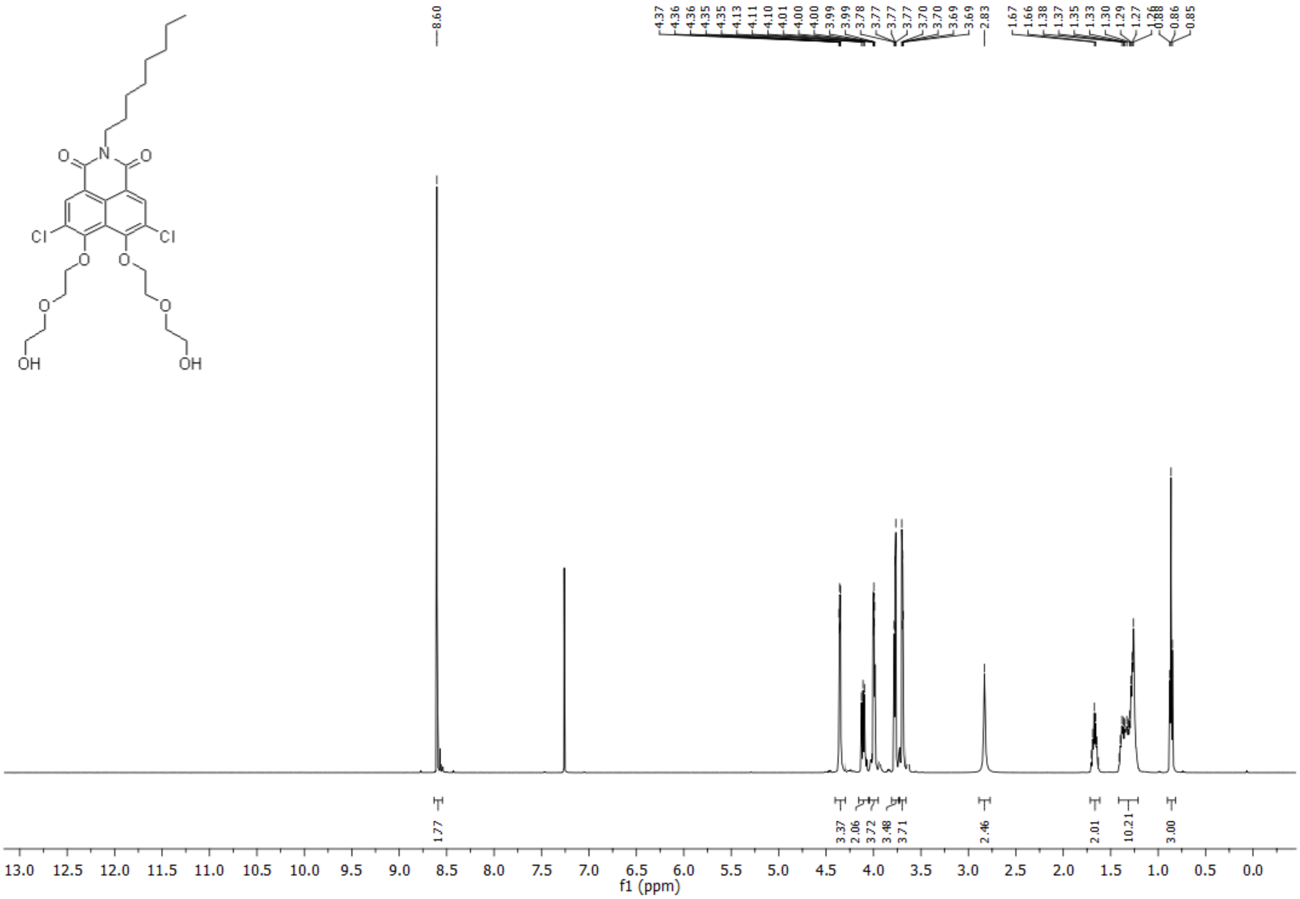

Figure S1: ^{1}H NMR spectrum of the compound **NI1**.

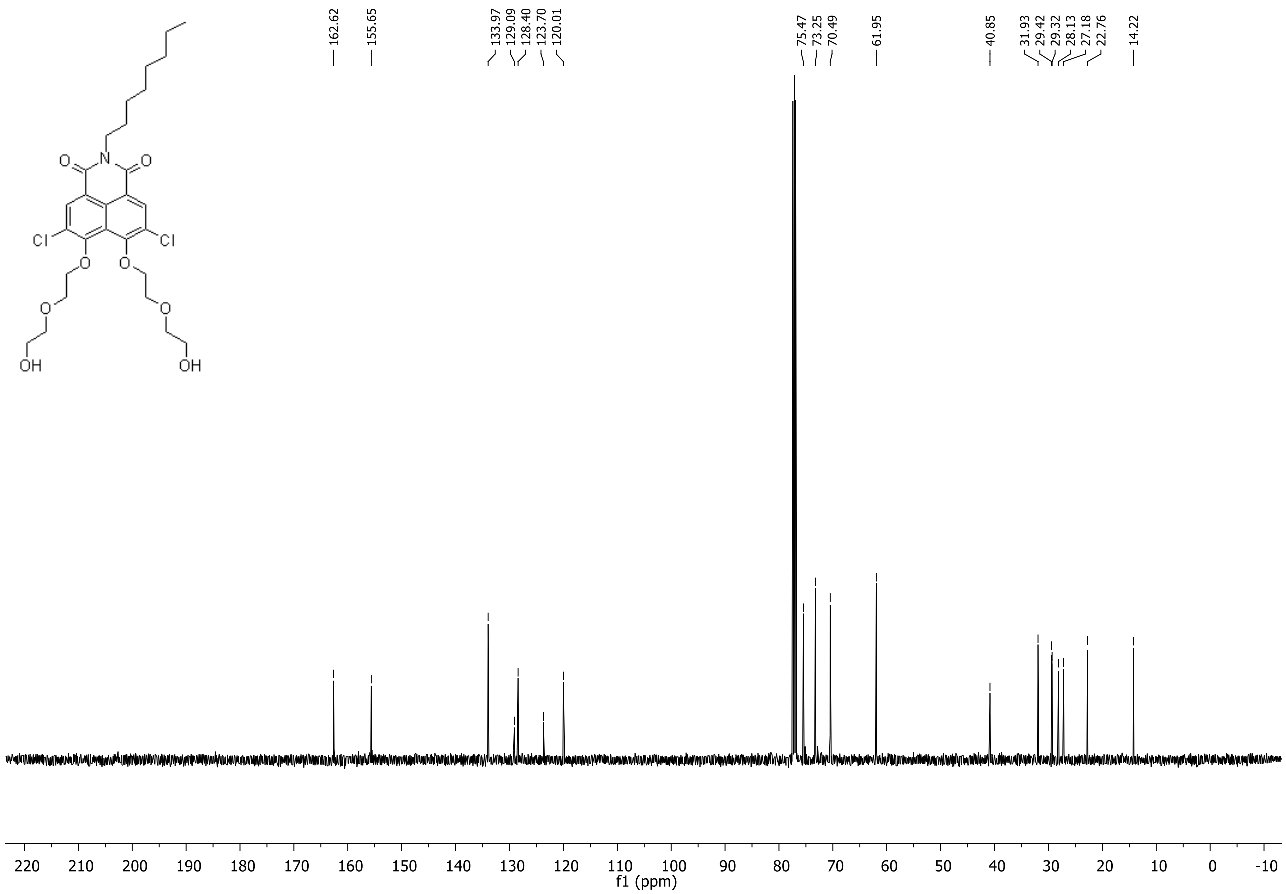

Figure S2: ^{13}C NMR spectrum of the compound **NI1**.

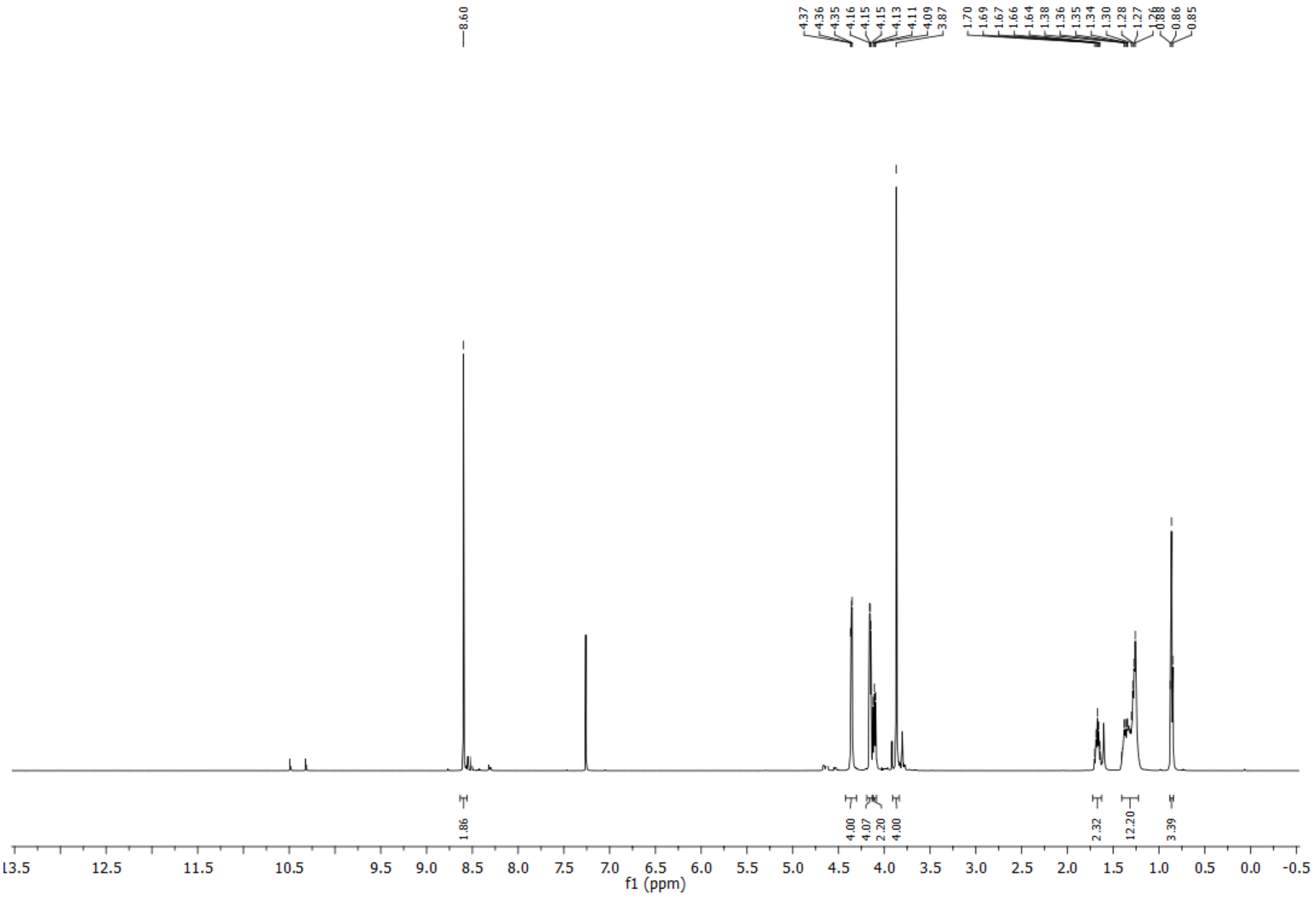

Figure S3: ^{1}H NMR spectrum of the compound **NI2.**

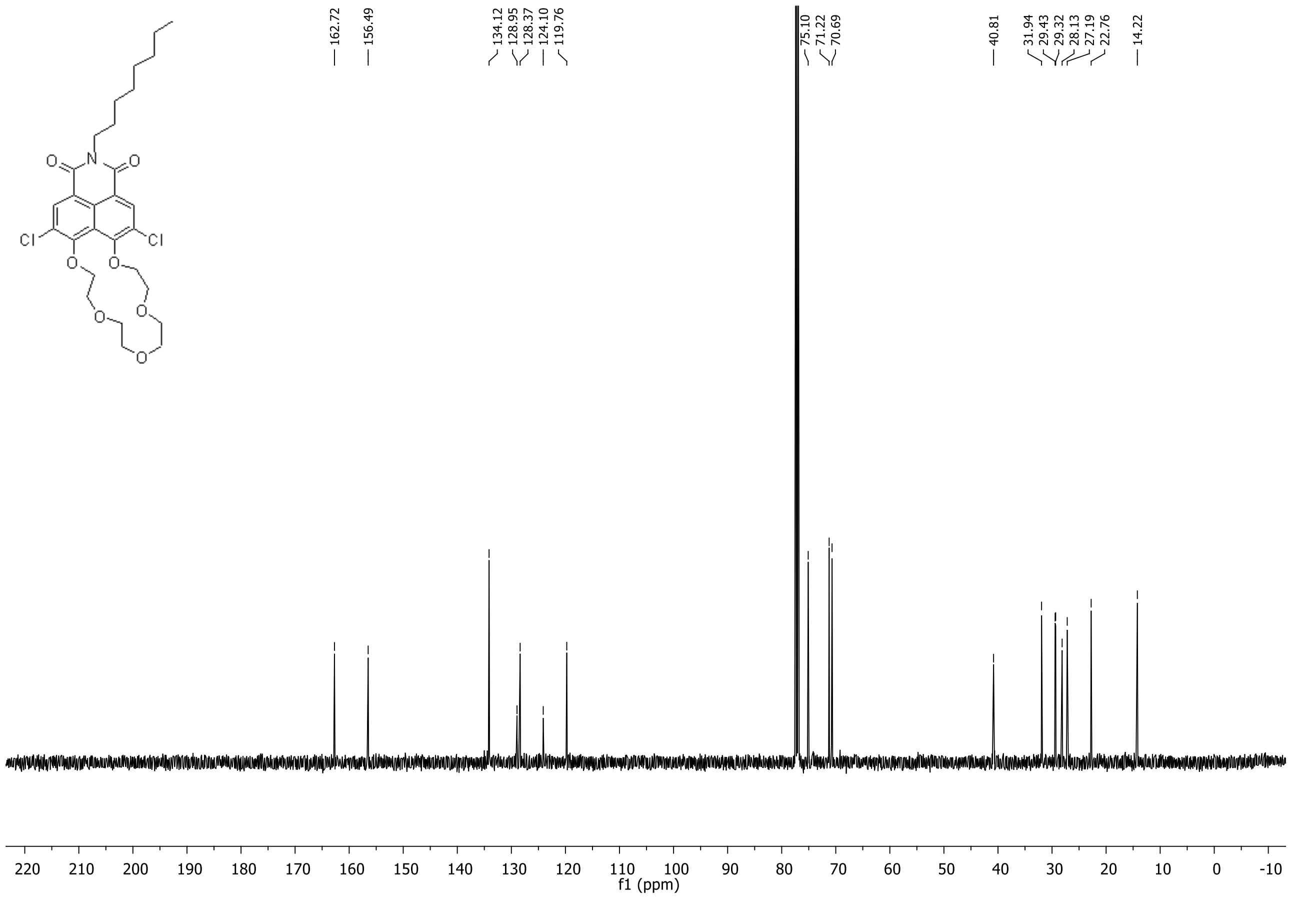

Figure S4: ^{13}C NMR spectrum of the compound **NI2.**

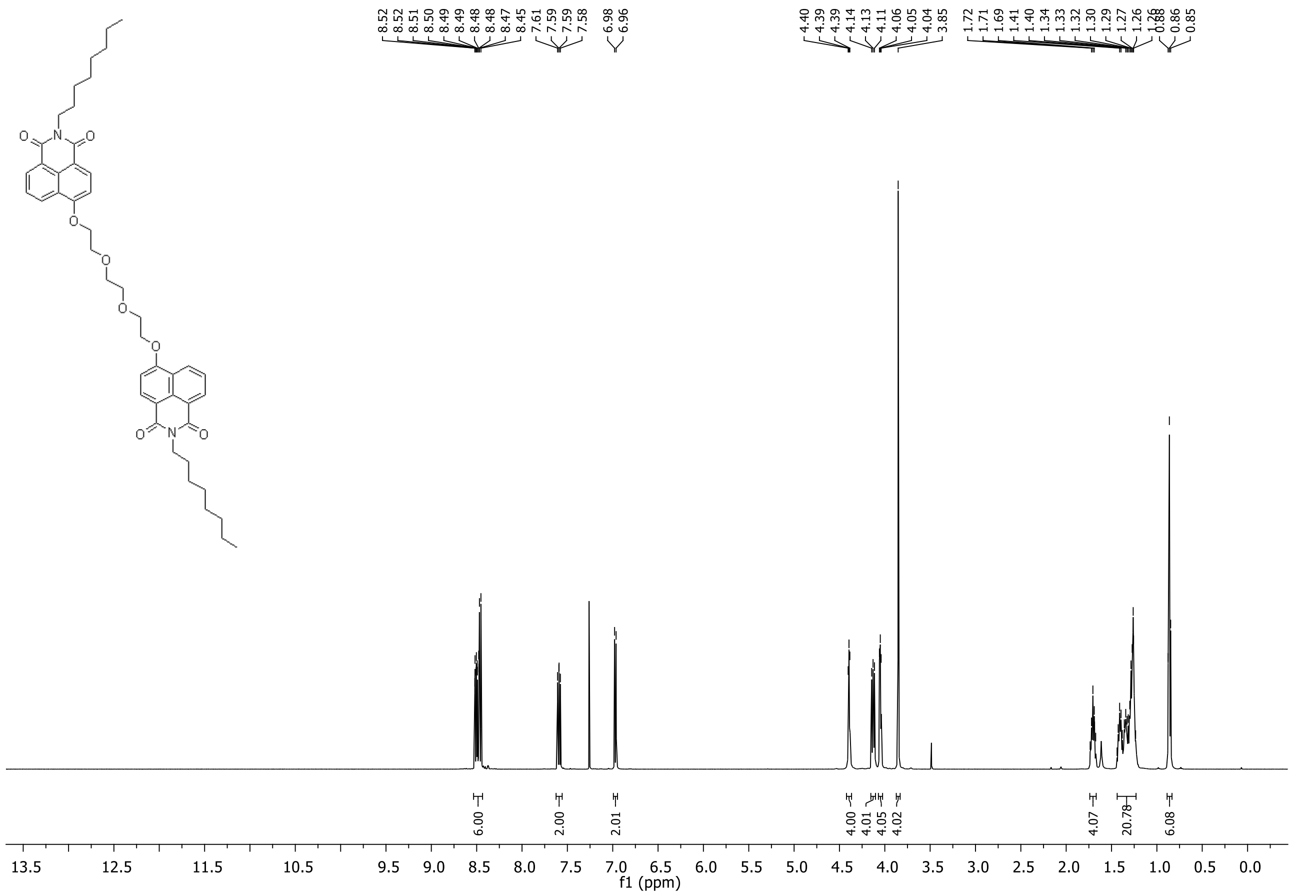

Figure S5: ^{1}H NMR spectrum of the compound **NI3**.

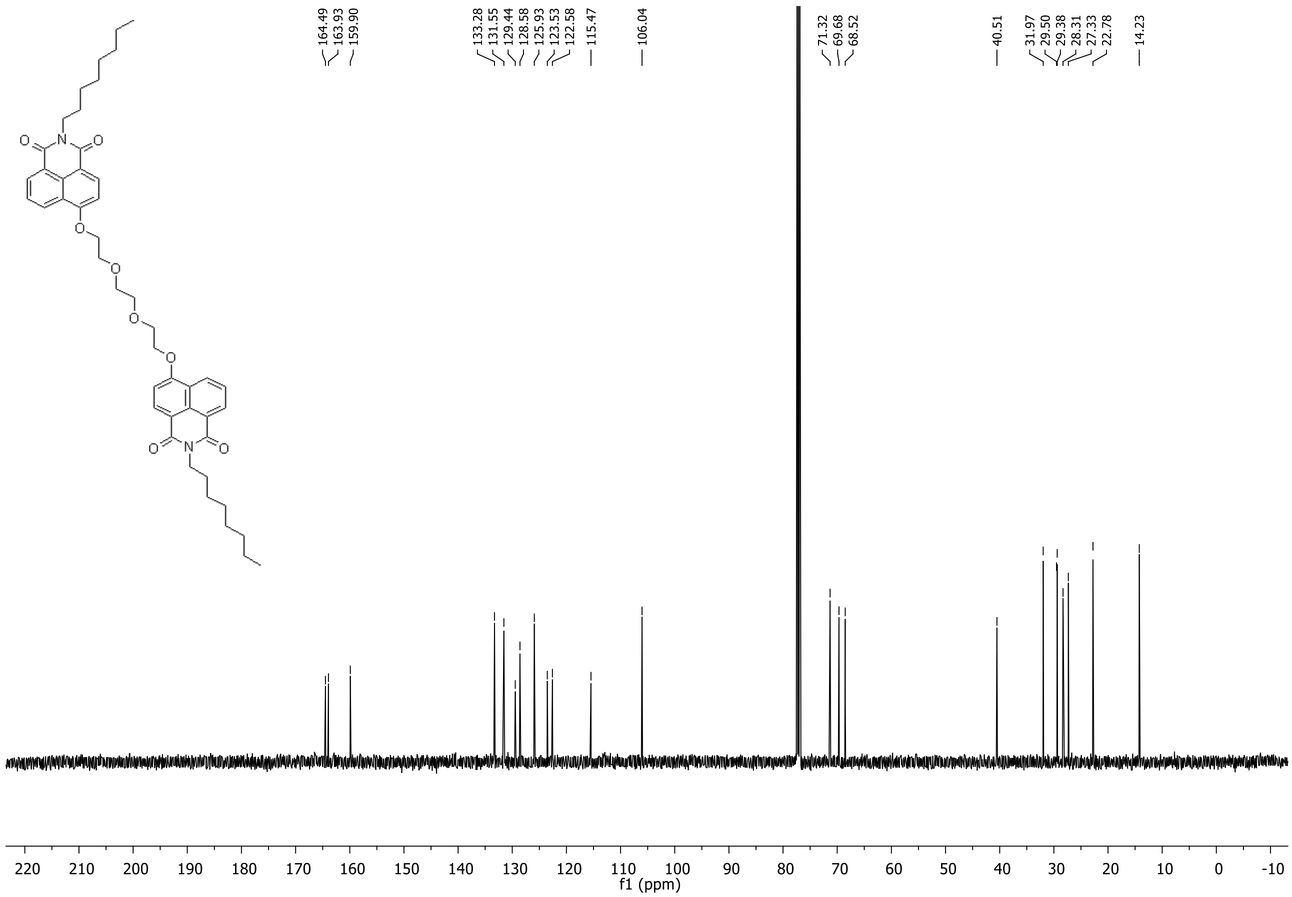

Figure S6: ^{13}C NMR spectrum of the compound **NI3**.

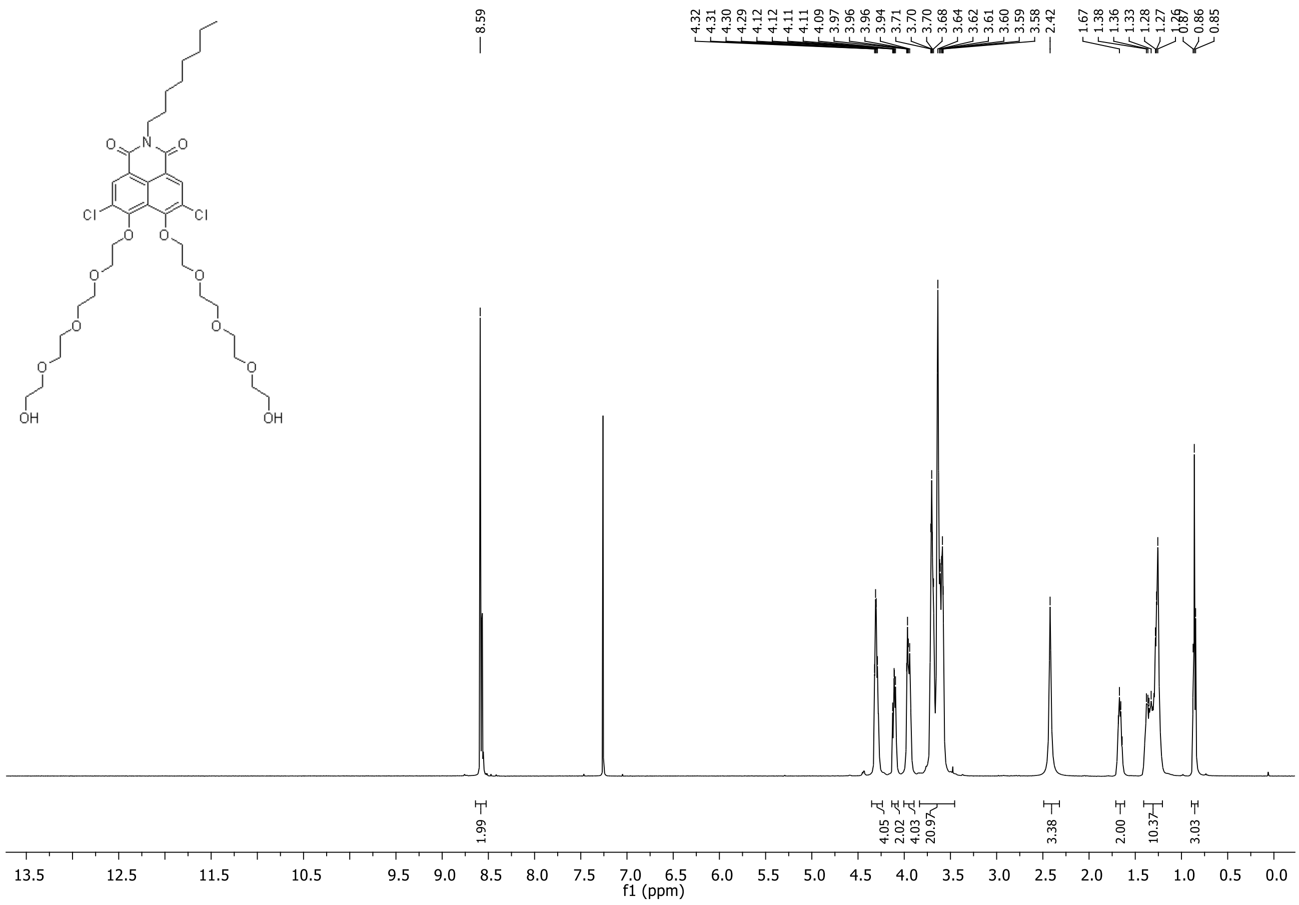

Figure S7: ^{1}H NMR spectrum of the compound **NI4.**

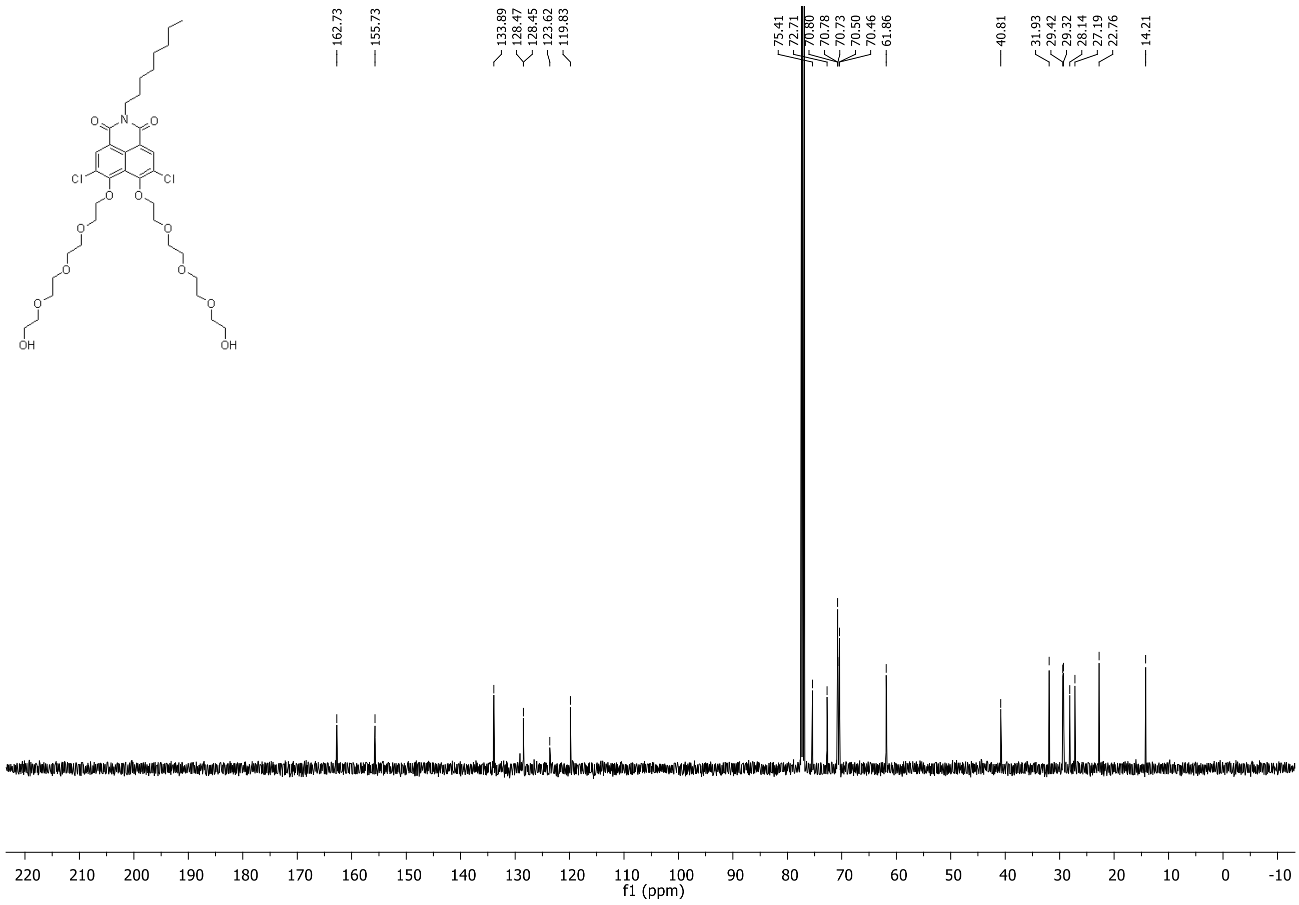

Figure S8: ^{13}C NMR spectrum of the compound **NI4**.

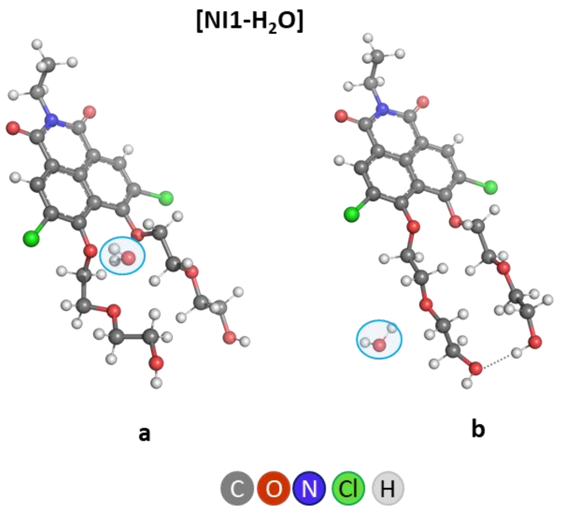

	ΔH, kcal mol[-1]		ΔH+BSSE, kcal mol[-1]	
	a	b	a	b
ε=1	0.00	0.14	0.47	0.00
ε=2	0.00	0.72	0.00	0.11
ε=5	0.00	0.99	0.00	0.38
ε=78	0.00	2.85	0.00	2.24

Figure S9: ωb97xd/6-311+G(d,p) optimized [NI1-H_2O](a) and [NI1-H_2O](b) complexes and relative stabilities (expressed as enthalpy difference ΔH).

Table S1: ωB97XD/6-311+G(d,p) optimized geometries of the studied compounds and/or their aggregates/complexes in the gas phase.

**Water dimer**			
8	1.504486000	-0.001282000	0.119708000
1	0.547827000	-0.000988000	-0.015671000
1	1.882120000	0.009293000	-0.759284000
8	-1.366169000	0.001123000	-0.101409000
1	-1.767132000	-0.767800000	0.306831000
1	-1.769348000	0.760761000	0.321736000
**[dmEG-H_2O]**			
6	0.801349000	-1.165705000	-0.051240000
6	-0.638139000	-1.023272000	-0.481222000
1	0.836358000	-1.486660000	0.999410000
1	1.284321000	-1.941329000	-0.664346000
1	-0.685794000	-0.486744000	-1.439730000
1	-1.065750000	-2.026600000	-0.626695000
8	1.477733000	0.060398000	-0.213258000
8	-1.368807000	-0.331559000	0.506469000
6	2.791893000	0.036488000	0.290340000
1	3.400377000	-0.719571000	-0.224292000
1	3.224531000	1.021367000	0.116558000
1	2.799617000	-0.173862000	1.367800000
6	-2.723173000	-0.163582000	0.151395000
1	-3.214899000	-1.133451000	-0.003348000
1	-3.212061000	0.355944000	0.975341000
1	-2.819746000	0.438363000	-0.761517000
1	0.464512000	1.815364000	-0.420350000
1	-0.778880000	1.717408000	0.382598000
8	-0.311946000	2.335686000	-0.187845000
**NI1**			
6	0.608137000	-1.193080000	-0.239811000
6	1.321979000	0.035354000	-0.069259000
6	0.685728000	1.300826000	0.133632000
6	1.313710000	-2.375475000	-0.351527000
6	2.740209000	-0.003011000	-0.103044000
6	3.416939000	-1.236278000	-0.223973000
6	2.713448000	-2.407008000	-0.328882000
6	4.900219000	-1.294687000	-0.244372000
6	4.972632000	1.167756000	-0.056646000
6	3.488561000	1.190980000	-0.012079000
6	2.856124000	2.397840000	0.128924000
6	1.459012000	2.442469000	0.212040000
1	3.443003000	3.304979000	0.201478000
1	3.246199000	-3.345145000	-0.422459000
8	5.502352000	-2.344185000	-0.304475000
8	5.634700000	2.179053000	0.025275000
6	7.049445000	-0.120006000	-0.224335000
1	7.383562000	0.796355000	-0.707650000

1	7.331159000	-0.974669000	-0.836811000
7	5.580350000	-0.078445000	-0.200706000
6	-1.590166000	-1.494112000	0.672468000
6	-2.959483000	-1.695430000	0.051764000
1	-1.603373000	-0.650493000	1.366959000
1	-1.244319000	-2.391394000	1.192246000
1	-3.104901000	-0.936833000	-0.731471000
1	-3.018909000	-2.684486000	-0.422925000
6	7.636719000	-0.239819000	1.175225000
1	7.349984000	0.617000000	1.788504000
1	7.296987000	-1.158233000	1.658464000
1	8.727307000	-0.268093000	1.117607000
8	-0.727853000	-1.190719000	-0.429877000
8	-3.938267000	-1.553553000	1.050208000
6	-5.238015000	-1.774330000	0.545694000
6	-6.267278000	-1.335227000	1.577945000
1	-5.381532000	-2.832036000	0.286519000
1	-5.382506000	-1.189300000	-0.374893000
1	-6.277234000	-2.028467000	2.421221000
1	-5.981300000	-0.348561000	1.968775000
8	-7.561449000	-1.327997000	1.026968000
1	-7.607379000	-0.617569000	0.373167000
6	-1.531326000	1.681792000	-0.690959000
1	-1.197606000	2.579025000	-1.219318000
1	-1.573616000	0.837279000	-1.382927000
6	-2.878946000	1.880459000	-0.024201000
1	-2.933366000	2.868507000	0.452796000
1	-2.992525000	1.119375000	0.759470000
6	-5.171749000	1.722202000	-0.400957000
1	-5.195231000	1.016734000	0.439836000
1	-5.424717000	2.719634000	-0.007512000
6	-6.188697000	1.276565000	-1.439283000
1	-5.831118000	0.367543000	-1.925368000
1	-6.324710000	2.037655000	-2.214059000
1	-7.882624000	1.743835000	-0.558550000
8	-0.638702000	1.375658000	0.384593000
8	-3.894090000	1.729534000	-0.988835000
8	-7.427238000	0.944255000	-0.825749000
17	0.454366000	-3.868253000	-0.598783000
17	0.692544000	3.980220000	0.489772000
**[NI1-H_2O]**			
6	0.781414000	-1.262285000	-0.354493000
6	1.424136000	-0.022413000	-0.048367000
6	0.715441000	1.168942000	0.299855000
6	1.554462000	-2.383027000	-0.589273000
6	2.841652000	0.028739000	-0.092962000
6	3.588831000	-1.140230000	-0.354084000
6	2.954277000	-2.333115000	-0.578588000
6	5.073135000	-1.105051000	-0.392330000
6	5.000118000	1.321538000	0.066028000
6	3.517927000	1.248299000	0.125945000
6	2.816108000	2.390850000	0.404615000
6	1.419989000	2.339868000	0.500843000

1	3.346958000	3.319514000	0.573012000
1	3.539105000	-3.223245000	-0.774480000
8	5.735334000	-2.102945000	-0.572701000
8	5.600055000	2.357026000	0.253427000
6	7.147797000	0.193816000	-0.267099000
1	7.413878000	1.175845000	-0.654246000
1	7.471127000	-0.570194000	-0.971711000
7	5.679401000	0.138845000	-0.220953000
6	-1.376448000	-1.791265000	0.539465000
6	-2.573893000	-2.479236000	-0.089558000
1	-1.701288000	-0.933002000	1.133843000
1	-0.808965000	-2.471416000	1.179839000
1	-2.839516000	-1.960793000	-1.021182000
1	-2.341567000	-3.522475000	-0.334411000
6	7.770056000	-0.034219000	1.103454000
1	7.438549000	0.729505000	1.810012000
1	7.504587000	-1.020662000	1.489219000
1	8.858472000	0.021300000	1.027699000
8	-0.554710000	-1.323144000	-0.536026000
8	-3.639210000	-2.409544000	0.833174000
6	-4.750227000	-3.207965000	0.460783000
6	-5.839805000	-3.009164000	1.503380000
1	-4.461585000	-4.265487000	0.409896000
1	-5.115757000	-2.895866000	-0.527793000
1	-5.503284000	-3.392495000	2.468275000
1	-6.045514000	-1.938079000	1.614859000
8	-7.005205000	-3.731263000	1.163306000
1	-7.435901000	-3.288822000	0.430407000
6	-1.510779000	1.572354000	-0.472859000
1	-1.029361000	2.312975000	-1.117036000
1	-1.805388000	0.710714000	-1.079117000
6	-2.707965000	2.171594000	0.237662000
1	-2.439798000	3.136214000	0.683952000
1	-3.030462000	1.501213000	1.047530000
6	-4.808090000	3.131145000	-0.250303000
1	-5.226280000	2.715673000	0.675573000
1	-4.442643000	4.147475000	-0.040624000
6	-5.878655000	3.165590000	-1.330257000
1	-6.293115000	2.165785000	-1.468568000
1	-5.429797000	3.483472000	-2.279298000
1	-6.671702000	4.912150000	-0.989471000
8	-0.611904000	1.135616000	0.549853000
8	-3.736659000	2.328681000	-0.714722000
8	-6.955389000	3.997988000	-0.951357000
17	0.781660000	-3.894494000	-0.968918000
17	0.569272000	3.792260000	0.943944000
8	-5.033506000	-0.221900000	-0.442423000
1	-4.479490000	-0.681364000	0.199320000
1	-4.540254000	0.565389000	-0.705635000
**NI2**			
6	-0.623214000	-1.053629000	0.053171000
6	0.168082000	0.138410000	0.069392000
6	-0.377352000	1.453422000	0.219561000

6	-0.001311000	-2.276274000	-0.114495000
6	1.575942000	0.008703000	-0.066099000
6	2.164315000	-1.264334000	-0.228830000
6	1.386326000	-2.391227000	-0.256793000
6	3.635033000	-1.415584000	-0.371635000
6	3.877739000	1.033497000	-0.165832000
6	2.403974000	1.152325000	-0.032274000
6	1.860776000	2.399044000	0.132002000
6	0.472708000	2.541251000	0.250673000
1	2.509415000	3.265676000	0.161703000
1	1.851324000	-3.360532000	-0.386135000
8	4.160501000	-2.500582000	-0.492806000
8	4.609000000	1.997717000	-0.111358000
6	5.851392000	-0.380752000	-0.492891000
1	6.209393000	0.521444000	-0.985577000
1	6.034825000	-1.239751000	-1.135846000
7	4.393153000	-0.246831000	-0.365260000
6	-2.500737000	-1.159138000	1.480274000
6	-3.924395000	-1.668815000	1.413555000
1	-2.458816000	-0.187268000	1.981318000
1	-1.892251000	-1.884283000	2.031762000
1	-3.970436000	-2.510705000	0.712335000
1	-4.183158000	-2.043492000	2.410712000
6	6.525404000	-0.562750000	0.860102000
1	6.336966000	0.299095000	1.503640000
1	6.162241000	-1.466802000	1.353340000
1	7.605334000	-0.658196000	0.725546000
8	-1.972032000	-1.016924000	0.152687000
8	-4.862614000	-0.670155000	1.081767000
6	-5.534962000	-0.793383000	-0.159666000
6	-2.449369000	1.736007000	-0.851755000
1	-2.111277000	2.612858000	-1.412521000
1	-2.267337000	0.840799000	-1.451173000
6	-3.926596000	1.825894000	-0.474608000
1	-4.256628000	2.865115000	-0.454025000
1	-4.053210000	1.403555000	0.524735000
6	-4.776996000	-0.234395000	-1.355567000
8	-1.703579000	1.646341000	0.372629000
8	-4.757805000	1.178939000	-1.418592000
17	-0.962833000	-3.727106000	-0.155591000
17	-0.189499000	4.136659000	0.443551000
1	-5.293796000	-0.553991000	-2.264466000
1	-3.763741000	-0.653635000	-1.377711000
1	-5.774703000	-1.848417000	-0.355730000
1	-6.474905000	-0.248364000	-0.044724000
**[NI2-H_2O]**			
6	-0.351123000	-1.188222000	0.137676000
6	0.338635000	0.062882000	0.104028000
6	-0.313516000	1.334271000	0.193468000
6	0.364282000	-2.363829000	0.026113000
6	1.752671000	0.039129000	-0.031986000
6	2.442096000	-1.189734000	-0.123944000
6	1.758851000	-2.375590000	-0.096632000

6	3.921912000	-1.230178000	-0.259108000
6	3.963275000	1.238146000	-0.215756000
6	2.485267000	1.244923000	-0.079930000
6	1.841202000	2.451184000	0.002457000
6	0.448164000	2.486776000	0.134422000
1	2.415501000	3.368622000	-0.033031000
1	2.300236000	-3.309845000	-0.177536000
8	4.532074000	-2.275607000	-0.309854000
8	4.613360000	2.259805000	-0.231288000
6	6.046483000	-0.029823000	-0.458199000
1	6.329356000	0.865010000	-1.009755000
1	6.299097000	-0.911042000	-1.045419000
7	4.582027000	-0.006891000	-0.332166000
6	-2.273684000	-1.415862000	1.516427000
6	-3.775075000	-1.517051000	1.384965000
1	-2.009437000	-0.548966000	2.127583000
1	-1.866396000	-2.329986000	1.958513000
1	-4.021953000	-2.107212000	0.493931000
1	-4.168346000	-2.046561000	2.264376000
6	6.733224000	-0.069051000	0.899956000
1	6.476701000	0.814966000	1.487541000
1	6.444510000	-0.965614000	1.452544000
1	7.817198000	-0.086222000	0.765105000
8	-1.706684000	-1.244189000	0.200804000
8	-4.323846000	-0.223029000	1.321055000
6	-5.522068000	-0.082749000	0.573321000
6	-2.468226000	1.513268000	-0.774913000
1	-1.965151000	2.123290000	-1.531017000
1	-2.612407000	0.505810000	-1.169715000
6	-3.795906000	2.133800000	-0.342450000
1	-3.784898000	3.211101000	-0.513400000
1	-3.910216000	1.957680000	0.729875000
6	-5.266139000	0.295216000	-0.877779000
8	-1.639018000	1.434030000	0.399369000
8	-4.901112000	1.650887000	-1.076488000
17	-0.481805000	-3.887446000	0.005915000
17	-0.338579000	4.032206000	0.246060000
1	-6.195200000	0.174389000	-1.440279000
1	-4.531691000	-0.392134000	-1.314512000
1	-6.109018000	-1.009782000	0.607061000
1	-6.104893000	0.701632000	1.064226000
8	-3.257144000	-2.280949000	-1.948344000
1	-2.589858000	-2.067479000	-1.283998000
1	-2.863798000	-2.952964000	-2.505329000
**NI3**			
6	-0.762749000	-0.545965000	1.844540000
6	-0.234040000	-1.639943000	1.093703000
6	-1.057335000	-2.632577000	0.520009000
6	0.074133000	0.425718000	2.347110000
6	1.162497000	-1.708511000	0.899387000
6	2.011157000	-0.727548000	1.460482000
6	1.463161000	0.320119000	2.157874000
6	3.475867000	-0.811882000	1.280670000

6	3.169656000	-2.841958000	-0.095130000
6	1.702461000	-2.752028000	0.118946000
6	0.877986000	-3.695287000	-0.447652000
6	-0.510541000	-3.638064000	-0.238683000
1	1.323165000	-4.481608000	-1.045877000
1	2.123715000	1.079300000	2.561196000
8	4.234976000	0.012081000	1.749788000
8	3.666496000	-3.710439000	-0.781170000
6	5.415092000	-1.993333000	0.353918000
1	5.664392000	-3.053759000	0.340701000
1	5.879324000	-1.526690000	1.220692000
7	3.962679000	-1.898797000	0.552617000
6	-2.749281000	0.520432000	2.643143000
6	-4.219900000	0.179903000	2.783200000
1	-2.612697000	1.430283000	2.049920000
1	-2.328565000	0.673695000	3.645091000
1	-4.319721000	-0.759761000	3.346453000
1	-4.690089000	0.975725000	3.366797000
6	5.869532000	-1.320018000	-0.933901000
1	5.408232000	-1.794770000	-1.800949000
1	5.598230000	-0.263723000	-0.938101000
1	6.955374000	-1.404463000	-1.026722000
8	-2.098170000	-0.567162000	2.001645000
8	-4.933368000	0.106756000	1.575162000
6	-4.761445000	-1.089430000	0.844114000
6	-5.931923000	-1.280412000	-0.102986000
1	-4.737811000	-1.948833000	1.532581000
1	-3.811428000	-1.068102000	0.303397000
1	-5.898585000	-2.306451000	-0.499919000
1	-6.859539000	-1.167568000	0.464380000
1	-1.149104000	-4.395857000	-0.677853000
1	-0.319035000	1.279205000	2.882143000
1	-2.125941000	-2.585157000	0.687362000
6	-5.438596000	-0.745644000	-2.381110000
6	-3.927105000	-0.918941000	-2.387150000
1	-5.888980000	-1.689412000	-2.727112000
1	-5.707730000	0.038954000	-3.090793000
1	-3.607783000	-1.774544000	-1.778436000
1	-3.595627000	-1.092555000	-3.418811000
8	-5.987073000	-0.343697000	-1.155196000
8	-3.369617000	0.270446000	-1.869676000
6	-2.039903000	0.381711000	-1.717633000
6	-1.626398000	1.537104000	-0.987426000
6	-1.110036000	-0.505095000	-2.213687000
6	-0.243796000	1.766299000	-0.822839000
6	-2.552941000	2.443814000	-0.426423000
6	0.260426000	-0.255941000	-2.029347000
1	-1.416081000	-1.393799000	-2.748725000
6	0.186940000	2.905745000	-0.111920000
6	0.698011000	0.858230000	-1.357117000
6	-2.108252000	3.545006000	0.265344000
1	-3.612072000	2.247300000	-0.541172000
1	0.995048000	-0.951679000	-2.417849000

6	-0.731189000	3.780078000	0.421323000
6	1.636302000	3.170648000	0.064235000
6	2.143801000	1.093167000	-1.182159000
1	-2.822284000	4.239137000	0.693021000
1	-0.369226000	4.648364000	0.959446000
7	2.521192000	2.242308000	-0.483157000
6	3.959648000	2.507815000	-0.336421000
6	4.514463000	3.274946000	-1.528106000
1	4.453156000	1.547319000	-0.217899000
1	4.083852000	3.072587000	0.584589000
1	5.582487000	3.456861000	-1.385707000
1	4.386184000	2.703370000	-2.450037000
1	4.015734000	4.241249000	-1.633885000
8	2.042870000	4.148455000	0.657097000
8	2.984146000	0.330041000	-1.616898000
**[NI3-H_2O]**			
6	-0.637730000	-0.689856000	1.767865000
6	-0.037858000	-1.741048000	1.010281000
6	-0.796186000	-2.761144000	0.396447000
6	0.134946000	0.314119000	2.305865000
6	1.364766000	-1.738235000	0.852616000
6	2.149000000	-0.725493000	1.450015000
6	1.532152000	0.282451000	2.148202000
6	3.620926000	-0.734161000	1.305917000
6	3.451982000	-2.765066000	-0.092154000
6	1.975826000	-2.743098000	0.073906000
6	1.214090000	-3.714405000	-0.531227000
6	-0.181241000	-3.725311000	-0.363370000
1	1.713187000	-4.469784000	-1.126668000
1	2.143067000	1.068524000	2.577398000
8	4.324436000	0.122477000	1.801509000
8	4.009866000	-3.608631000	-0.761941000
6	5.640853000	-1.818997000	0.428563000
1	5.940796000	-2.866292000	0.454519000
1	6.054045000	-1.306392000	1.295061000
7	4.179881000	-1.789062000	0.582037000
6	-2.697600000	0.213498000	2.596118000
6	-4.117739000	-0.278810000	2.790528000
1	-2.676484000	1.151351000	2.030485000
1	-2.252726000	0.377486000	3.585346000
1	-4.097981000	-1.258905000	3.286403000
1	-4.634287000	0.425843000	3.446359000
6	6.105316000	-1.162195000	-0.864285000
1	5.702630000	-1.688056000	-1.730898000
1	5.778816000	-0.122948000	-0.914185000
1	7.196787000	-1.190966000	-0.915613000
8	-1.975124000	-0.787001000	1.894209000
8	-4.894053000	-0.348134000	1.614048000
6	-4.616979000	-1.443842000	0.758332000
6	-5.749259000	-1.605229000	-0.239412000
1	-4.548554000	-2.364585000	1.356417000
1	-3.660639000	-1.291971000	0.253266000
1	-5.658406000	-2.589605000	-0.719489000

1	-6.700456000	-1.576551000	0.298010000
1	-0.770159000	-4.504201000	-0.833909000
1	-0.313974000	1.141375000	2.838523000
1	-1.870145000	-2.768787000	0.533481000
6	-5.197938000	-0.860723000	-2.456667000
6	-3.683811000	-0.983882000	-2.419404000
1	-5.606753000	-1.782948000	-2.895495000
1	-5.471740000	-0.025888000	-3.104218000
1	-3.356953000	-1.875671000	-1.868671000
1	-3.320475000	-1.072437000	-3.451088000
8	-5.809006000	-0.586980000	-1.218809000
8	-3.176900000	0.178077000	-1.801438000
6	-1.844435000	0.339365000	-1.677969000
6	-1.451701000	1.491827000	-0.932027000
6	-0.899817000	-0.507717000	-2.213702000
6	-0.071441000	1.749158000	-0.780359000
6	-2.391096000	2.368236000	-0.343076000
6	0.467011000	-0.228964000	-2.043651000
1	-1.187974000	-1.393690000	-2.762641000
6	0.343745000	2.880272000	-0.047150000
6	0.885405000	0.876809000	-1.347451000
6	-1.958886000	3.458958000	0.373559000
1	-3.454642000	2.179597000	-0.448116000
1	1.212630000	-0.897408000	-2.458147000
6	-0.586323000	3.719549000	0.521579000
6	1.787719000	3.175896000	0.116090000
6	2.328265000	1.135775000	-1.175643000
1	-2.687185000	4.126526000	0.819140000
1	-0.235858000	4.581866000	1.076772000
7	2.687972000	2.277196000	-0.457729000
6	4.121266000	2.571451000	-0.318688000
6	4.654155000	3.351719000	-1.511843000
1	4.635063000	1.621335000	-0.203428000
1	4.237816000	3.137098000	0.602623000
1	5.718846000	3.555794000	-1.374340000
1	4.533193000	2.779648000	-2.434408000
1	4.134817000	4.307625000	-1.613003000
8	2.180217000	4.151711000	0.721297000
8	3.179947000	0.395249000	-1.628309000
1	-5.419118000	1.338402000	0.803354000
8	-5.640785000	2.006078000	0.142390000
1	-5.885253000	1.449467000	-0.603452000
**NI4**			
6	-3.741372000	-1.146341000	0.414421000
6	-4.471369000	0.034195000	0.068054000
6	-3.849618000	1.268649000	-0.301103000
6	-4.429741000	-2.315522000	0.673252000
6	-5.888767000	-0.022817000	0.091588000
6	-6.548212000	-1.240668000	0.366135000
6	-5.828360000	-2.375048000	0.634470000
6	-8.030716000	-1.320632000	0.375095000
6	-8.135987000	1.096900000	-0.126820000
6	-6.652363000	1.136942000	-0.164548000

6	-6.034773000	2.323541000	-0.459927000
6	-4.637502000	2.377879000	-0.539391000
1	-6.632980000	3.204164000	-0.658304000
1	-6.346822000	-3.302394000	0.843878000
8	-8.617784000	-2.364086000	0.559935000
8	-8.812164000	2.078969000	-0.341520000
6	-10.195802000	-0.188578000	0.192550000
1	-10.545816000	0.768284000	0.576367000
1	-10.469305000	-0.977261000	0.890985000
7	-8.727415000	-0.130167000	0.170673000
6	-1.514737000	-1.522870000	-0.401164000
6	-0.161518000	-1.594388000	0.278266000
1	-1.505433000	-0.786291000	-1.208003000
1	-1.822918000	-2.495149000	-0.794726000
1	-0.035215000	-0.705367000	0.911671000
1	-0.114937000	-2.480718000	0.926224000
6	-10.776459000	-0.462479000	-1.187833000
1	-10.504355000	0.331700000	-1.885992000
1	-10.417582000	-1.418826000	-1.574349000
1	-11.866407000	-0.506584000	-1.127745000
8	-2.410420000	-1.091169000	0.628543000
8	0.845803000	-1.634555000	-0.702403000
6	2.120978000	-1.768371000	-0.118293000
6	3.177079000	-1.567556000	-1.183345000
1	2.237489000	-2.760297000	0.339693000
1	2.258480000	-1.016001000	0.669727000
1	3.051496000	-2.303157000	-1.990456000
1	3.085043000	-0.563424000	-1.620107000
8	4.424016000	-1.723205000	-0.551831000
6	-1.636188000	1.801706000	0.457699000
1	-1.992705000	2.756323000	0.853852000
1	-1.572868000	1.067994000	1.264418000
6	-0.295527000	1.936965000	-0.239636000
1	-0.257919000	2.866741000	-0.823967000
1	-0.182097000	1.096736000	-0.938680000
6	2.000666000	1.959950000	0.109604000
1	2.085936000	1.172790000	-0.651994000
1	2.153933000	2.928357000	-0.386460000
6	3.066518000	1.749167000	1.163247000
1	2.917357000	0.778594000	1.656640000
1	3.008063000	2.530670000	1.933626000
8	-2.525599000	1.321173000	-0.556448000
8	0.731665000	1.906012000	0.720006000
8	4.305712000	1.788728000	0.499888000
17	-3.550021000	-3.745423000	1.131701000
17	-3.889850000	3.872983000	-1.022224000
6	5.381828000	1.369714000	1.306676000
6	6.662193000	1.691487000	0.563953000
1	5.313698000	0.290255000	1.500646000
1	5.375512000	1.895202000	2.271593000
1	6.660826000	1.201600000	-0.420317000
1	6.732617000	2.776076000	0.400663000
6	8.991154000	1.512759000	0.757149000

1	9.055214000	1.044555000	-0.236167000
1	9.123596000	2.595244000	0.619572000
6	5.510803000	-1.396460000	-1.382700000
6	6.777359000	-1.726789000	-0.620883000
1	5.489968000	-0.328253000	-1.640157000
1	5.477927000	-1.973969000	-2.317449000
1	6.802125000	-1.178653000	0.330932000
1	6.804097000	-2.802461000	-0.397572000
6	9.108043000	-1.656176000	-0.827054000
1	9.171680000	-1.203249000	0.171107000
1	9.236160000	-2.741159000	-0.708298000
6	10.087255000	0.964746000	1.661414000
1	9.835247000	-0.065276000	1.947301000
1	10.136400000	1.552928000	2.580293000
6	10.204163000	-1.083581000	-1.704739000
1	9.933647000	-0.062197000	-1.996527000
1	10.319571000	-1.683616000	-2.612725000
8	7.740166000	1.231515000	1.341097000
8	7.873306000	-1.356371000	-1.426164000
8	11.401873000	-1.087851000	-0.934466000
1	12.155399000	-0.986217000	-1.516491000
8	11.348438000	1.042176000	1.040652000
1	11.421304000	0.316335000	0.406334000
**[NI4-H_2O]**			
6	-3.806470000	-1.127053000	0.581138000
6	-4.535585000	0.015032000	0.121833000
6	-3.916190000	1.223335000	-0.329754000
6	-4.493924000	-2.268759000	0.944097000
6	-5.953508000	-0.054310000	0.113976000
6	-6.611389000	-1.244474000	0.493756000
6	-5.891231000	-2.340206000	0.890110000
6	-8.093011000	-1.338192000	0.474871000
6	-8.204513000	1.009277000	-0.286605000
6	-6.720579000	1.065452000	-0.275356000
6	-6.106578000	2.227038000	-0.662346000
6	-4.708328000	2.294955000	-0.693803000
1	-6.707842000	3.076325000	-0.962227000
1	-6.409088000	-3.245815000	1.180439000
8	-8.676826000	-2.361070000	0.758795000
8	-8.881184000	1.953661000	-0.629773000
6	-10.261436000	-0.267665000	0.090249000
1	-10.638304000	0.728521000	0.314627000
1	-10.557082000	-0.952839000	0.882804000
7	-8.794106000	-0.186609000	0.120388000
6	-1.613775000	-1.573654000	-0.248023000
6	-0.221025000	-1.487288000	0.338888000
1	-1.696083000	-0.950824000	-1.142558000
1	-1.876986000	-2.607873000	-0.487541000
1	-0.017513000	-0.450108000	0.634146000
1	-0.157850000	-2.111639000	1.240750000
6	-10.774261000	-0.749931000	-1.260067000
1	-10.480153000	-0.058962000	-2.052882000
1	-10.384612000	-1.744610000	-1.485851000

1	-11.865142000	-0.806538000	-1.243311000
8	-2.472337000	-1.061351000	0.775748000
8	0.707042000	-1.915341000	-0.630225000
6	2.005504000	-1.478055000	-0.320950000
6	3.002290000	-2.132506000	-1.254170000
1	2.264486000	-1.726264000	0.718619000
1	2.077355000	-0.387252000	-0.436401000
1	3.070699000	-3.209353000	-1.057052000
1	2.705447000	-1.984861000	-2.300177000
8	4.238701000	-1.499226000	-0.989891000
6	-1.777293000	1.859964000	0.522383000
1	-2.245351000	2.769782000	0.907750000
1	-1.669205000	1.132961000	1.331014000
6	-0.426318000	2.140102000	-0.111265000
1	-0.418642000	3.122614000	-0.600668000
1	-0.241233000	1.379765000	-0.884086000
6	1.844756000	2.383927000	0.405371000
1	2.012627000	1.918894000	-0.575507000
1	1.962083000	3.469229000	0.286204000
6	2.863733000	1.846047000	1.391547000
1	2.726313000	0.762218000	1.502197000
1	2.732734000	2.309374000	2.377847000
8	-2.580799000	1.292865000	-0.518514000
8	0.559010000	2.067447000	0.892076000
8	4.148148000	2.130742000	0.876695000
17	-3.615590000	-3.646680000	1.541504000
17	-3.960676000	3.758024000	-1.266070000
6	5.184596000	1.423559000	1.529443000
6	6.502201000	1.902268000	0.952593000
1	5.068551000	0.343874000	1.360546000
1	5.157877000	1.611106000	2.610418000
1	6.509593000	1.744194000	-0.136234000
1	6.620767000	2.978713000	1.139035000
6	8.817030000	1.543566000	1.127489000
1	8.909959000	1.382340000	0.043819000
1	8.996605000	2.610853000	1.316940000
6	5.374370000	-2.140335000	-1.537418000
6	6.555762000	-1.257604000	-1.190769000
1	5.271120000	-2.252397000	-2.624073000
1	5.503572000	-3.134815000	-1.091871000
1	6.476953000	-0.300464000	-1.725030000
1	6.535789000	-1.043828000	-0.111941000
6	8.865642000	-1.172981000	-1.144805000
1	8.906639000	-0.221945000	-1.695843000
1	8.802693000	-0.942124000	-0.073763000
6	9.849854000	0.693283000	1.859634000
1	9.492391000	-0.344762000	1.893528000
1	9.948263000	1.034139000	2.892639000
6	10.128961000	-1.963110000	-1.403660000
1	10.243057000	-2.158436000	-2.475213000
1	10.080887000	-2.919515000	-0.871610000
8	7.530324000	1.168594000	1.569616000
8	7.746249000	-1.925602000	-1.534552000

8	11.197191000	-1.156664000	-0.918207000
1	12.027974000	-1.620897000	-1.020041000
8	11.118459000	0.793347000	1.259701000
1	11.143519000	0.208569000	0.491014000
8	4.210561000	1.233271000	-1.871856000
1	4.189645000	0.314563000	-1.573586000
1	4.250742000	1.736017000	-1.049179000
**3*NI1**			
6	1.788381000	-3.895396000	1.686339000
6	1.344057000	-4.063296000	0.331060000
6	0.066682000	-4.640535000	-0.035929000
6	3.050392000	-3.380103000	1.929093000
6	2.245254000	-3.688690000	-0.701560000
6	3.526281000	-3.178301000	-0.401359000
6	3.927043000	-3.033731000	0.899449000
6	4.506579000	-2.871280000	-1.474527000
6	2.841974000	-3.557138000	-3.151484000
6	1.881638000	-3.831605000	-2.057424000
6	0.654757000	-4.336215000	-2.380734000
6	-0.246695000	-4.735547000	-1.386736000
1	0.394825000	-4.462364000	-3.424466000
1	4.918965000	-2.659583000	1.118331000
8	5.601522000	-2.410530000	-1.228116000
8	2.550747000	-3.696582000	-4.318183000
6	5.153219000	-3.130860000	-3.834397000
1	4.924566000	-3.954614000	-4.510176000
1	6.108344000	-3.319636000	-3.347016000
7	4.130912000	-3.177594000	-2.776662000
6	0.180433000	-3.164441000	3.186432000
6	-0.875122000	-3.726320000	4.115055000
1	-0.296902000	-2.692471000	2.324654000
1	0.775874000	-2.403428000	3.695395000
1	-1.384787000	-4.558552000	3.619285000
1	-0.425874000	-4.106919000	5.039937000
6	5.197044000	-1.819907000	-4.602299000
1	4.215359000	-1.587587000	-5.020470000
1	5.539722000	-1.002140000	-3.967944000
1	5.902808000	-1.914718000	-5.431148000
8	1.021534000	-4.229113000	2.745790000
8	-1.770836000	-2.669012000	4.384899000
6	-3.116904000	-3.057074000	4.551463000
6	-3.942385000	-1.791346000	4.745625000
1	-3.223677000	-3.734926000	5.412252000
1	-3.486047000	-3.582791000	3.659657000
1	-3.762370000	-1.359807000	5.736990000
1	-3.638764000	-1.048004000	4.006722000
8	-5.320956000	-2.030363000	4.522266000
1	-5.668967000	-2.543512000	5.251810000
6	-2.088611000	-5.163993000	1.064553000
1	-2.525005000	-5.815611000	0.313287000
1	-2.275363000	-5.604103000	2.043561000
6	-2.683801000	-3.773311000	0.995318000
1	-2.489743000	-3.312439000	0.017342000

1	-2.234719000	-3.141630000	1.771321000
6	-4.707843000	-2.635811000	1.178303000
1	-4.402190000	-2.029233000	2.041178000
1	-4.408793000	-2.099721000	0.271711000
6	-6.218788000	-2.834543000	1.158703000
1	-6.608196000	-2.892180000	2.175433000
1	-6.438400000	-3.776696000	0.645800000
1	-6.829557000	-1.914296000	-0.453708000
8	-0.661060000	-5.119097000	0.980915000
8	-4.069651000	-3.895828000	1.206566000
8	-6.868011000	-1.754065000	0.508942000
17	3.594323000	-3.186250000	3.569165000
17	-1.721062000	-5.458606000	-1.983864000
6	1.565366000	-0.062363000	1.145633000
6	2.149682000	0.060552000	-0.151744000
6	1.396966000	0.012404000	-1.361429000
6	2.360706000	0.113589000	2.259860000
6	3.546991000	0.269207000	-0.244060000
6	4.333813000	0.350988000	0.922570000
6	3.747195000	0.287754000	2.156325000
6	5.803308000	0.549523000	0.840019000
6	5.618953000	0.704514000	-1.603757000
6	4.163212000	0.429455000	-1.500418000
6	3.424101000	0.352237000	-2.650062000
6	2.047423000	0.111466000	-2.573447000
1	3.902839000	0.496179000	-3.608552000
1	4.358074000	0.371309000	3.046094000
8	6.491885000	0.597767000	1.836968000
8	6.148859000	0.923925000	-2.671135000
6	7.791441000	0.958531000	-0.517994000
1	8.136323000	0.506932000	-1.446313000
1	8.256816000	0.450141000	0.323956000
7	6.350400000	0.695135000	-0.424860000
6	-0.730363000	0.540777000	1.515602000
6	-1.937378000	-0.259194000	1.967643000
1	-0.916182000	1.089860000	0.588373000
1	-0.417881000	1.230723000	2.300719000
1	-2.222658000	-0.960294000	1.172019000
1	-1.657273000	-0.843965000	2.846851000
6	8.091507000	2.451132000	-0.488981000
1	7.629305000	2.950544000	-1.342102000
1	7.709473000	2.902298000	0.428376000
1	9.171070000	2.612531000	-0.536172000
8	0.278124000	-0.447758000	1.284430000
8	-3.027269000	0.545177000	2.335226000
6	-3.954274000	0.846444000	1.312094000
6	-5.333956000	0.971946000	1.955408000
1	-3.992081000	0.031370000	0.578544000
1	-3.670925000	1.767907000	0.786124000
1	-5.428230000	0.192341000	2.719740000
1	-5.436327000	1.938870000	2.453777000
8	-6.372515000	0.856082000	1.003591000
1	-6.589221000	-0.092083000	0.888660000

6	-0.635649000	-1.237440000	-1.497118000
1	-0.180263000	-1.825806000	-2.295218000
1	-0.595792000	-1.800193000	-0.559862000
6	-2.047294000	-0.839656000	-1.853152000
1	-2.058987000	-0.344939000	-2.834309000
1	-2.406750000	-0.108023000	-1.112378000
6	-4.181596000	-1.598984000	-2.218813000
1	-4.586915000	-0.880798000	-1.488666000
1	-4.158592000	-1.096432000	-3.190296000
6	-5.118513000	-2.791537000	-2.272124000
1	-4.927216000	-3.459355000	-1.428605000
1	-4.969323000	-3.365203000	-3.192629000
1	-6.560861000	-1.557519000	-2.742489000
8	0.045832000	0.005786000	-1.333760000
8	-2.873429000	-1.974725000	-1.859744000
8	-6.459838000	-2.338667000	-2.169523000
17	1.650497000	0.103180000	3.846249000
17	1.133663000	-0.002986000	-4.047268000
6	-1.279985000	3.919984000	0.751275000
6	-0.044979000	3.752719000	0.048034000
6	0.059563000	3.704159000	-1.377333000
6	-1.277978000	3.916518000	2.132870000
6	1.149519000	3.650017000	0.809420000
6	1.103790000	3.685352000	2.219616000
6	-0.093543000	3.805152000	2.872208000
6	2.348383000	3.624843000	3.029233000
6	3.659450000	3.462807000	0.947445000
6	2.399695000	3.530658000	0.166309000
6	2.477650000	3.507311000	-1.200305000
6	1.305807000	3.584314000	-1.961799000
1	3.443983000	3.425211000	-1.681408000
1	-0.112832000	3.825584000	3.954567000
8	2.330151000	3.694258000	4.236205000
8	4.742143000	3.383249000	0.405865000
6	4.789548000	3.406958000	3.117838000
1	5.467852000	2.740929000	2.588343000
1	4.524255000	2.955006000	4.071910000
7	3.548522000	3.485160000	2.331763000
6	-2.759553000	5.413283000	-0.320315000
6	-4.222083000	5.731976000	-0.092042000
1	-2.497767000	5.499988000	-1.376791000
1	-2.150462000	6.112912000	0.262128000
1	-4.483965000	5.526812000	0.954266000
1	-4.354042000	6.801877000	-0.275570000
6	5.420433000	4.775940000	3.324852000
1	5.662205000	5.237581000	2.365441000
1	4.748047000	5.432113000	3.881554000
1	6.346128000	4.671152000	3.894807000
8	-2.459357000	4.067237000	0.105915000
8	-5.104945000	5.077415000	-0.965463000
6	-5.387176000	3.733153000	-0.646109000
6	-6.687428000	3.361593000	-1.344574000
1	-5.483183000	3.603999000	0.441686000

1	-4.585421000	3.066106000	-0.983958000
1	-7.533229000	3.853619000	-0.850756000
1	-6.650761000	3.728151000	-2.373660000
8	-6.866813000	1.963591000	-1.390332000
1	-6.721166000	1.596909000	-0.495642000
6	-1.701107000	2.612528000	-2.514302000
1	-0.972133000	1.815957000	-2.679219000
1	-2.362255000	2.331256000	-1.687047000
6	-2.483796000	2.903582000	-3.787115000
1	-1.783689000	3.099757000	-4.600284000
1	-3.098002000	3.802175000	-3.633105000
6	-4.467856000	1.662533000	-3.434859000
1	-4.277837000	1.165693000	-2.474986000
1	-4.894916000	2.648220000	-3.215895000
6	-5.457473000	0.826431000	-4.242675000
1	-4.906878000	0.039817000	-4.764807000
1	-5.935306000	1.448753000	-5.008146000
1	-6.718261000	0.850097000	-2.747648000
8	-1.025408000	3.829507000	-2.170773000
8	-3.281740000	1.813680000	-4.184388000
8	-6.426024000	0.206036000	-3.419995000
17	-2.776827000	4.083795000	2.994671000
17	1.440057000	3.574160000	-3.691904000
**[NI1-H_2O] (b)**			
6	0.824108000	-1.272911000	-0.282277000
6	1.490035000	-0.035259000	-0.013559000
6	0.807605000	1.177267000	0.318454000
6	1.575449000	-2.407810000	-0.517983000
6	2.907329000	-0.008043000	-0.079867000
6	3.631942000	-1.193944000	-0.328864000
6	2.975597000	-2.379762000	-0.527485000
6	5.115789000	-1.184997000	-0.384037000
6	5.089433000	1.250380000	0.030466000
6	3.606834000	1.204343000	0.106450000
6	2.928543000	2.364448000	0.371295000
6	1.532955000	2.340139000	0.486350000
1	3.478963000	3.285775000	0.515740000
1	3.544343000	-3.281282000	-0.718468000
8	5.759353000	-2.197066000	-0.553598000
8	5.709488000	2.278305000	0.192823000
6	7.214133000	0.076319000	-0.293968000
1	7.498135000	1.048943000	-0.691804000
1	7.518866000	-0.701679000	-0.991638000
7	5.745279000	0.050684000	-0.240094000
6	-1.332976000	-1.751203000	0.649698000
6	-2.708516000	-1.966468000	0.047252000
1	-1.365994000	-0.971877000	1.415198000
1	-0.930966000	-2.671279000	1.081688000
1	-2.906742000	-1.159175000	-0.673637000
1	-2.738486000	-2.918816000	-0.499585000
6	7.836326000	-0.150943000	1.076871000
1	7.526525000	0.629400000	1.775163000
1	7.547087000	-1.125771000	1.474896000

1	8.925573000	-0.124474000	0.997636000
8	-0.515677000	-1.312283000	-0.441543000
8	-3.664963000	-1.942858000	1.076265000
6	-4.965722000	-2.203660000	0.591385000
6	-5.986919000	-1.894608000	1.677823000
1	-5.057019000	-3.248158000	0.264396000
1	-5.170066000	-1.568196000	-0.283128000
1	-5.937842000	-2.646540000	2.467815000
1	-5.741035000	-0.925161000	2.133553000
8	-7.293802000	-1.915443000	1.159494000
1	-7.397215000	-1.153706000	0.573242000
6	-1.440290000	1.537757000	-0.421265000
1	-1.157586000	2.498086000	-0.862815000
1	-1.461145000	0.764476000	-1.193317000
6	-2.780918000	1.607927000	0.284770000
1	-2.877774000	2.543410000	0.850187000
1	-2.845960000	0.771095000	0.990476000
6	-5.083046000	1.348351000	-0.063928000
1	-5.028096000	0.568598000	0.703899000
1	-5.380723000	2.289921000	0.418722000
6	-6.096578000	0.938224000	-1.120743000
1	-5.705102000	0.091717000	-1.688253000
1	-6.285196000	1.759042000	-1.817894000
1	-7.832381000	1.249188000	-0.254507000
8	-0.513788000	1.169984000	0.601939000
8	-3.812685000	1.495882000	-0.675580000
8	-7.297960000	0.494077000	-0.503086000
17	0.773258000	-3.908107000	-0.882425000
17	0.708796000	3.810447000	0.918822000
8	-4.643219000	3.776660000	-2.098859000
1	-4.127522000	3.023367000	-1.779575000
1	-4.165182000	4.127178000	-2.849968000
